# Genome-wide analyses of member identification, expression pattern, and protein–protein interaction of *EPF/EPFL* gene family in *Gossypium*

**DOI:** 10.1186/s12870-024-05262-7

**Published:** 2024-06-14

**Authors:** Pengtao Li, Zilin Zhao, Wenkui Wang, Tao Wang, Nan Hu, Yangyang Wei, Zhihao Sun, Yu Chen, Yanfang Li, Qiankun Liu, Shuhan Yang, Juwu Gong, Xianghui Xiao, Yuling Liu, Yuzhen Shi, Renhai Peng, Quanwei Lu, Youlu Yuan

**Affiliations:** 1 School of Biotechnology and Food Engineering, Anyang Institute of Technology, Anyang , Henan, 455000 China; 2 National Key Laboratory of Cotton Bio-breeding and Integrated Utilization, Institute of Cotton Research of Chinese Academy of Agricultural Sciences, Anyang, Henan 455000 China; 3 College of Agriculture, Tarim University, Alaer , Xinjiang, 843300 China

**Keywords:** Cotton, *EPF/EPFL* gene family, Expression pattern, Protein–protein interaction, qRT-PCR verification

## Abstract

**Background:**

Epidermal patterning factor / -like (*EPF/EPFL*) gene family encodes a class of cysteine-rich secretory peptides, which are widelyfound in terrestrial plants.Multiple studies has indicated that EPF/EPFLs might play significant roles in coordinating plant development and growth, especially as the morphogenesis processes of stoma, awn, stamen, and fruit skin. However, few research on *EPF/EPFL* gene family was reported in *Gossypium*.

**Results:**

We separately identified 20 *G. raimondii*, 24 *G. arboreum*, 44 *G. hirsutum*, and 44 *G. barbadense EPF/EPFL* genes in the 4 representative cotton species, which were divided into four clades together with 11 *Arabidopsis thaliana*, 13 *Oryza sativa*, and 17 *Selaginella moellendorffii* ones based on their evolutionary relationships. The similar gene structure and common motifs indicated the high conservation among the EPF/EPFL members, while the uneven distribution in chromosomes implied the variability during the long-term evolutionary process. Hundreds of collinearity relationships were identified from the pairwise comparisons of intraspecifc and interspecific genomes, which illustrated gene duplication might contribute to the expansion of cotton *EPF/EPFL* gene family. A total of 15 kinds of cis-regulatory elements were predicted in the promoter regions, and divided into three major categories relevant to the biological processes of development and growth, plant hormone response, and abiotic stress response. Having performing the expression pattern analyses with the basic of the published RNA-seq data, we found most of *GhEPF/EPFL* and *GbEPF/EPFL* genes presented the relatively low expression levels among the 9 tissues or organs, while showed more dramatically different responses to high/low temperature and salt or drought stresses. Combined with transcriptome data of developing ovules and fibers and quantitative Real-time PCR results (qRT-PCR) of 15 highly expressed *GhEPF/EPFL* genes, it could be deduced that the cotton *EPF/EPFL* genes were closely related with fiber development. Additionally, the networks of protein–protein interacting among EPF/EPFLs concentrated on the cores of GhEPF1 and GhEPF7, and thosefunctional enrichment analyses indicated that most of EPF/EPFLs participate in the GO (Gene Ontology) terms of stomatal development and plant epidermis development, and the KEGG (Kyoto Encyclopedia of Genes and Genomes) pathways of DNA or base excision repair.

**Conclusion:**

Totally, 132 *EPF/EPFL* genes were identified for the first time in cotton, whose bioinformatic analyses of cis-regulatory elements and expression patterns combined with qRT-PCR experiments to prove the potential functions in the biological processes of plant growth and responding to abiotic stresses, specifically in the fiber development. These results not only provide comprehensive and valuable information for cotton *EPF/EPFL* gene family, but also lay solid foundation for screening candidate *EPF/EPFL* genes in further cotton breeding.

**Supplementary Information:**

The online version contains supplementary material available at 10.1186/s12870-024-05262-7.

## Background

In the process of plant growth and development, information must be transmitted between cells to ensure the basic life activities, among which protein secretion is one of the important ways of cell–cell communication. There are two common pathways of protein secretion, and the classical ER-Golgi pathway aims at the N-terminal of proteins with signal peptide sequences, meanwhile depends on endoplasmic (ER) and Golgi apparatus. The other non-classical pathway aims at the same N-terminal of proteins while without signal peptide sequences, nevertheless is independent of ER and Golgi apparatus [1]. The proteolytic enzymes, also namely as protease in plants, are generally consisted of these secreted proteins. They could break substrates into small peptide segments by hydrolyzing peptide bonds, which are divided into cystein protease, serine protease, aspartate protease, and metalloprptease based on their different catalytic sites [2]. Among the total 500–800 kinds of plant proteases encoded by *Arabidopsis thaliana* genome, cystein proteases (CPs) could contribute approximately 140 kinds, and are consisted of five protein families, such as papain proteinases (family C1), vacuolar processing enzyme (family VPE), caspases (family C14), calcium-dependent proteinases (family C2), and other CP family [3]. Given that CPs presented the different expression patterns in diverse tissues and organs, the previous reports have proved that CPs could play important roles in affecting the biological processes, such as seed germination, root growth, leaf senescence, and programmed cell death (PCD) of tracheary elements in stems and of tapetum cells in anthers [4–8]. These CPs makes significant differences in the specific positions through protein degradation for providing nutrients and components for the development of new cells, ensuring that the biological processes involved in plant growth proceed smoothly.

Epidermal patterning factor / -like (EPF/EPFL) proteins are one of the cysteine-rich plant-specific secreted peptides, which normally have 6 or 8 conserved cysteine residues in the C-terminal of peptide chain. These cysteine residues could form inter-molecular disulfide bond so as to affect the folding of peptide chain and protein activity [9]. Besides the C-terminal signal peptide, there is 1 alpha helix, 2 reverse beta folds, and 1 irregular ring region connecting with 2 beta folds, forming the core skeleton of EPF/EPFL peptides. Among the peptides, the 2 beta folds constitute the active region of the molecular, while the irregular ring region takes the responsibility for molecular specificity [10]. EPF/EPFL genes are ubiquitous in terrestrial plants, such as *Physcomotrella patens*, *Selaginella moellendorffii*, *Picea glauca*, *Sorghum bicolor*, *Populus trichocarpa*, *Medicago truncatula*, *Carica papaya*, while their genome-wide family analyses were only reported on *A. thaliana*, *Oryza sativa,* and *Malus domestica* [11–13]. During the specific stages of plant growth and development, EPF1 and EPF2 were found to combine with 3 LRR-RLKs (Leucine-rich repeat receptor kinases), namely as ERECTA (ER), ERECTA-LIKE1 (ERL1), and ERL2, and to interact with 1 LRR-receptor-like protein (LRR-RLP), namely as TOO MANY MOUTHS (TMM), collectively transmitting extracellular specific stomatal development signals [14–17]. In addition, the homologous *EPFL1* was reported to induce the awn elongation in rice [18]. Meanwhile, the ectopic expression of wheat *EPFL1* in *A. thaliana* resulted in the shorter filaments and peduncles, implying its correlation with flower development [19]. Gene editing experiments conducted in Kasalath (one rice cultivar) to knockout 11 *OsEPF/EPFL* genes by CRISPR/Cas9 (Clustered regularly interspaced short palindromic repeats) indicated that *OsEPFL2* participated in the regulation of awn development [20], which was also found to combine with ER family receptor-kinases for promoting leaf margin morphogenesis with adequate auxin supply in *A. thaliana* [21]. The *EPF/EPFL* genes involved in the inflorescence development, including *EPFL4*, *EPFL5*, and *EPFL6* [22–24], and those genes controlling the stomata development in stem were found as *EPFL4* and *EPFL6* [22]. EPFL9*,* also known as STOMAGEN, was mainly expressed in the mesophyll tissue below the epidermis. EPFL9 was observed to competed with EPFL1 and EPFL2 to combine with ER family receptors, which played an antagonistic role to promote the stomata formation in spite of not inducing the activation of phosphorylation of downstream signaling components [25, 26]. Furthermore, over-expressed *EPF1* was capable of improving the water-use efficiency (WUE) of poplar, barley, and rice, afterwards promoting the drought resistance of plants [27–29]. As well, over-expressed *EPF2* in *A. thaliana* reduced the density of stomata, which made the plants more tolerant to drought stress, while never produced adversely influences on the absorption of nitrogen or phosphorous [30, 31].

The plentiful studies relevant to *EPF/EPFL* gene family proved their potentially significant functions in affecting plant development and growth, especially as the morphogenesis processes of stoma, awn, stamen, and fruit skin [30, 31]. However, there was few relative researches reported in *Gossypium* species. Cotton is one of the most important industrial crops widely planted in all over the world, which provides the abundant fiber materials for textile industries, meanwhile produces plenty of vegetable proteins and edible oils for animals and humans [32, 33]. As the two most concerned agronomic traits in cotton, fiber yield and quality are of great significance for national welfare and the people’s livelihood. While, fiber yield and quality suffer a variety of influencing factors related to plant development and growth [34, 35]. What’s more, the total number of 53 *Gossypium* species are consisted of 46 diploid and 7 allotetraploid subspecies, of which the former could be divided into A-G and K chromosome types based on their similarities and differences of relationship and geographical distribution, while the latter AADD genomes were deduced to undergo hybridization between the allogenetic genome ancestors of diploid AA and DD together with polyploidization [36–39]. The accurate genome information and different ploidy characteristics are conducive to comprehensive analysis of cotton gene family that might participate in the vital biological processes. Therefore, it is of necessity and significance to perform genome-wide identification of *EPF/EPFL* gene family in cotton. With the combinations of expression pattern and protein–protein interaction of EPF/EPFLs referred to the public data, this study screened some candidate genes that might play key roles in regulating the plant development and growth. These results provides abundant gene resources for further cultivating novel cotton varieties with high yield, superior fiber quality, and multiple resistance by high-efficiency and precise genetic transformation technology.

## Materials and methods

### Genome-wide identification and physiochemical predictions of cotton EPF/EPFL family

The protein sequences of 11 EPF/EPFLs in*A A. thaliana* [11] were firstly downloaded from Ensembl Plants (http://plants.ensembl.org/index.html), of which EPF1, EPF2, and EPFL1-8 had the conserved domain of EPF (PF17181), while EPFL9 harbored the conserved domain of Stomagen (PF16851). These protein sequences were subsequently utilized as queries to BLASTP and hmmsearch (hidden markov model) in the four representative cotton genomes by TBtools [40], including *G. arboreun* (A2) [36], *G. raimondid* (D5) [37], *G. hirsutum* (AD1) [38], and *G. barbadense* (AD2) [38] obtained from CottonFGD (https://cotto nfgd.net/). The protein sequences of 13 EPF/EPFLs in *O. sativa* and 17 EPF/EPFLs in *S. moellendorffii* were separately downloaded from Rice Annotation Project database and the Phytozome v8.0 [11]. After removing the redundant sequences, the identified cotton EPF/EPFLs were further confirmed as the candidate proteins using Pfam (http://pfam.xfam.org./) and NCBI-CDD (http://www.ncbi.nlm.nih.gov/cdd) databases. The genome-wide members of cotton *EPF/EPFL* gene family were separately identified from the diploid and tetraploid genomes, which were named based their chromosomal locations and homologous relationships with *AtEPF/EPFL* genes. Meanwhile, the physiochemical characteristics of cotton *EPF/EPFL* genes were also predicted by the on-line tool ProParam (http://web.expasy.org/protparam/) [41], including the length of amino acids (AA), molecular weight (MW), isoelectric point (IP), and instability index. Besides, the subcellular localization of cotton EPF/EPFLs was predicted by WoLF PSORT (http://wolfpsort.hgc.jp/) [42].

## Construction of phylogenetic tree of EPF/EPFL proteins

The software Clustal X was chosen to perform the multiple sequence alignment on the 132 cotton, 11*A* AtEPF/EPFL, 13 OsEPF/EPFL, and 17 SmEPF/EPFL proteins [43], which were subsequently utilized to construct the phylogenetic tree using the neighbor-joining method of the software MEGA7.0 [44]. The Bootstrap value as the calibration parameter was set as 1000, and the drawing model was selected as the p-distance. At last, on-line tool Evolview (http://evolgenius.info //evolview-v2/#login) was used to modify the evolution tree [45].

## Mapping *EPF/EPFL* genes in cotton chromosomes

The software TBtools was chosen in this study to extract the position information of *EPF/EPFL* genes in the chromosomes according to the genome sequences and annotation files of the four representative cotton species. Thesoftware MapChart (https://help.salesforce.com/s/articleView?id=sf.bi_chart_intro_ map.htm&type = 5) was utilized to draw and visualize the physical locations in the cotton chromosomes.

## Analyses of gene structure and conserved protein motif of cotton EPF/EPFL genes

Firstly, the annotation files of cotton *EPF/EPFL* genes were entered into Gene Structure Display Server (GSDS, http://gsds.cbi.pku.edu.cn) so as to analyze their exon–intron structures [46]. Subsequently, the on-line website MEME SUITE (http://meme-suite.org/memei) was utilized for identifying the conserved motifs in cotton EPF/EPFL proteins [47]. Finally, the software TBtools was used to perform the visual-merge mapping on the phylogenetic tree, gene structure, and conserved protein motif of all the cotton EPF/EPFL family members.

## Collinearity analysis of cotton *EPF/EPFL* genes

The gene sequences of cotton EPF/EPFL family was subjected to collinearity analysis by the MCScanX software [48], whose visualization was displayed by the software TBtools. The collinearity analyses were composed of intraspecific and interspecific BLAST, which were separately conducted on the diploid genomes of A2 (*G. arboretum*) and D5 (*G. raimondii*) and the allotetraploid genomes of AD1 (*G. hirsutum*) and AD2 (*G. barbadense*). Subsequently, the duplicated gene pairs were identified from the intraspecific collinearity as Ga-Ga, Gr-Gr, Gh-Gh, and Gb-Gb, which were also identified from the interspecific collinearity as Ga-Gr, Ga-Gh, Ga-Gb, Gr-Gh, Gr-Gb, and Gh-Gb. The obtained duplication events were finally presented as collinearity relationships with the intraspecific and interspecific covariance circles.

## Analyses of *cis*-regulatory elementsof cotton *EPF/EPFL* genes

The DNA sequences of 2000 bp upstream of initiation codon (ATG) of all the 132 cotton *EPF/EPFL* genes were downloaded as their promoter regions from CottonFGD (http://cottonfgd.net/) database, and on-line tool PlantCARE (http://bioinformatics.psb. ugent.be/webtools/plantcare/html/) [49] was chosen to perform prediction analysis of cis-regulatory elements. The visualization of the predicted cis-regulatory elements were shown by TBtools, and the colorful rectangles presented the different cis-regulatory elements with the same clades of evolution relationships.

## Analyses of expression patterns and quantitative Real-time PCR verification

The transcriptome data of *G. hirsutum* TM-1 and *G. barbadense* Hai7124 on the different tissues/organs (root, stem, leaf, petal, torus, sepal, epicalyx, anther, and pistill) were downloaded from the SRA (Sequence Read Archive) database of NCBI website (http://www.ncbi.nlm.nih.gov/, and the accession number was PRJNA490626). Meanwhile, the transcriptome data of *G. hirsutum* TM-1 in response to multiple abiotic stresses (low temperature at 4℃, high temperature at 37℃, salt treatment of 0.4 M NaCl, and drought treatment of 200 g/liter PEG6000) were also obtained from the SRA database under the accession number PRJNA248163 [32]. The filtering treatment was firstly carried out on the published RNA-seq data by Trimmomatic software [50], and the obtained clean data were subsequently subjected to mapping on the reference genome databases built by the HISAT software [51]. The software Cufflinks was chosen to calculate the expression levels of cotton *EPF/EPFL* genes with the presentation of FPKM (fragments per kilobase of transcript per million fragments) values [52], which were utilized to show whether to express or not, and to show high or low expression levels of all the *GhEPF/EPFL* and *GbEPF/EPFL* genes in the different tissues/organs. The FPKM values of all the *GhEPF/EPFL* genes under the different adversity stresses were subjected to uniformization treatment by the Z-score algorithm, which was performed in order to investigate their up-regulated and down-regulated expression patterns along with the processes of stress occurrence and proceeding [53]. The heat-map of expression levels and patterns of cotton *EPF/EPFL* genes was finally drawn by the softwareTBtools.

The transcriptome data of *G. hirsutum* TM-1 and *G. barbadense* Hai7124 on the developing ovules (0, 1, 3, 5, 10, and 20 days after anthesis, DPA) and fibers (10, 20, and 25 DPA) were downloaded from the SRA database under the accession number of PRJNA490626. These data were subjected to the same treatments in turn as the aforesaid descriptions, including filtering the low-quality data, mapping to the reference genome, calculating the expression level, and Z-score uniformization. The positive and negative values represented the up-regulated and down-regulated expression patterns during the development of ovules and fibers, respectively, whose heat-map was also drawn by the software TBtools. In addition, two cultivated species, namely *G. hirsutum* CCRI36 and *G. barbadense* Hai1, were chosen in this study to perform quantitative Real-time PCR (qRT-PCR) experiment. The CCRI36 harbored the merits of high yield and wide adaptability, while subjected to normal fiber quality and low resistance to *Verticillium* wilt, and the Hai1 had the characteristics of superior fiber quality, high VW-resistance, low yield, and poor adaptability [54, 55]. In 2022, CCRI36 and Hai1 were planted in the experimental field of Anyang Institute of Technology (Anyang, Henan province), and the fiber samples were separately collected with three biological repeats at 10, 20, and 25 DPA after the flowers were in advance tagged on the day of anthesis (recorded as 0 DPA). The fiber samples were orderly subjected to RNA extraction with RNAprep Pure Plant Kit (Tiangen, Beijing, China), quality test with agarose gel electrophoresis and NanoDrop 2000 (Agilent Technologies, CA, USA), and cDNA synthesis with TranScript All-in-One First-Strand cDNA Synthesis SuperMix ofr qPCR kit (Transgen Biotech, Beijing, China). According to the detailed protocol of TransStart Top Green qPCR SuperMix kit (Transgen Biotch, Beijing, China), qRT-PCR experiment of 15 high expressed *GhEPF/EPFL* genes with the specific primers (Additional file 1: Table S1) was carried out on the ABI 7500fast Real-time PCR System (Applied Biosystems, USA) followed the PCR procedures at 20 μL volume as: 94℃ for 30 s (1 cycle); 94℃ for 5 s and 60℃ for 34 s (40 cycles); 60℃ for 60 s (1 cycle). The house-keeping gene *Ubiquitin7* (*UB7)* was utilized as the internal reference for normalizing the relative expression levels, which was calculated by the 2^−ΔΔCt^ method [56].

## Analyses of protein–protein interaction and functional enrichment of *GhEPF/EPFL* genes

In consideration of the fact that there was no protein data of cotton species recorded into the STRING database (http://string-db.org), homologous alignment was firstly performed between the 44 GhEPF/EPFLs and 11 AtEPF/EPFLs, of which the protein sequences of the latter ones were inputted as the protein models into STRING database for constructing the protein–protein interacting network [57]. Meanwhile, the 44 *GhEPF/EPFL* genes were also subjected to functional enrichment by GO (Gene Ontology) and KEGG (Kyoto Encyclopedia of Genes and Genomes) databases, of which the former was generally divided into three categories, namely biological process, cellular component, and molecular function [58], and the latter was composed of cellular processes, environmental information processing, genetic information processing, human disease, metabolism, and organismal systems [59]. The enrichment analyses of GO and KEGG were accomplished by the on-line platform OmicShare (https://www.omicshare.com/), and the detailed procedures were as followed: the ID numbers of GO and KEGG of all the cotton genes were firstly extracted from the released annotation information of *G. hirsutum* TM-1 [32], which were separately inputted into the OmicShare tools of GO Enrichment Analysis Advanced and Pathway Enrichment Analysis Advanced as the background files together with the 44 *GhEPF/EPFL* ids as the queries, finally generating the classification results.

## Results

### Identification and physiochemical characteristic analyses of cotton *EPF/EPFL* genes

Twenty-four *GaEPF/EPFL*, 20 *GrEPF/EPFL*, 44 *GhEPF/EPFL*, and 44 *GbEPF/EPFL* genes were separately identified from the genomes of *G. arboreum* (A2), *G. raimondii* (D5), *G. hirsutum* (AD1) and *G. barbadense* (AD2) [33, 36, 37], reaching the total number of 132 members (Additional file 1: Table S2). Meanwhile, the analyses of physico-chemical properties of cotton EPF/EPFL family showed that the lengths of amino acid sequences of EPF/EPFL proteins ranged from 87 aa (*GrEPF20* and *GhEPF35*) to 161 aa (*GhEPF36* and *GbEPF35*), and those molecular weight ranges were from 9760.13 kDa (*GrEPF20*) to 18,049.99 kDa (*GhEPF36* and *GbEPF35*). The isoelectric points of cotton EPF/EPFLs ranged from 5.53 (GhEPF15 and GbEPF17) to 9.91 (GhEPF44), whose average value was 8.84. The ranges of instability index of cotton EPF/EPFLs were from 30.62 (GhEPF21) to 84.76 (GhEPF5), including 12 possibly stable members (≦ 40) and 120 possibly unstable members (> 40). The results of subcellular localization prediction showed that most of cotton EPF/EPFL genes were located in chloroplast (59 members) and extracellular (51 members), and the minority ones were located in mitochondria (9 members), endoplasmic reticulum (4 members), nucleus (4 members), vacuole (4 members), and plasmalemma (1 member).

## Phylogenetic analysis of EPF/EPFL proteins in *Gossypium* and *Arabidopsis*

**Fig. 1:**
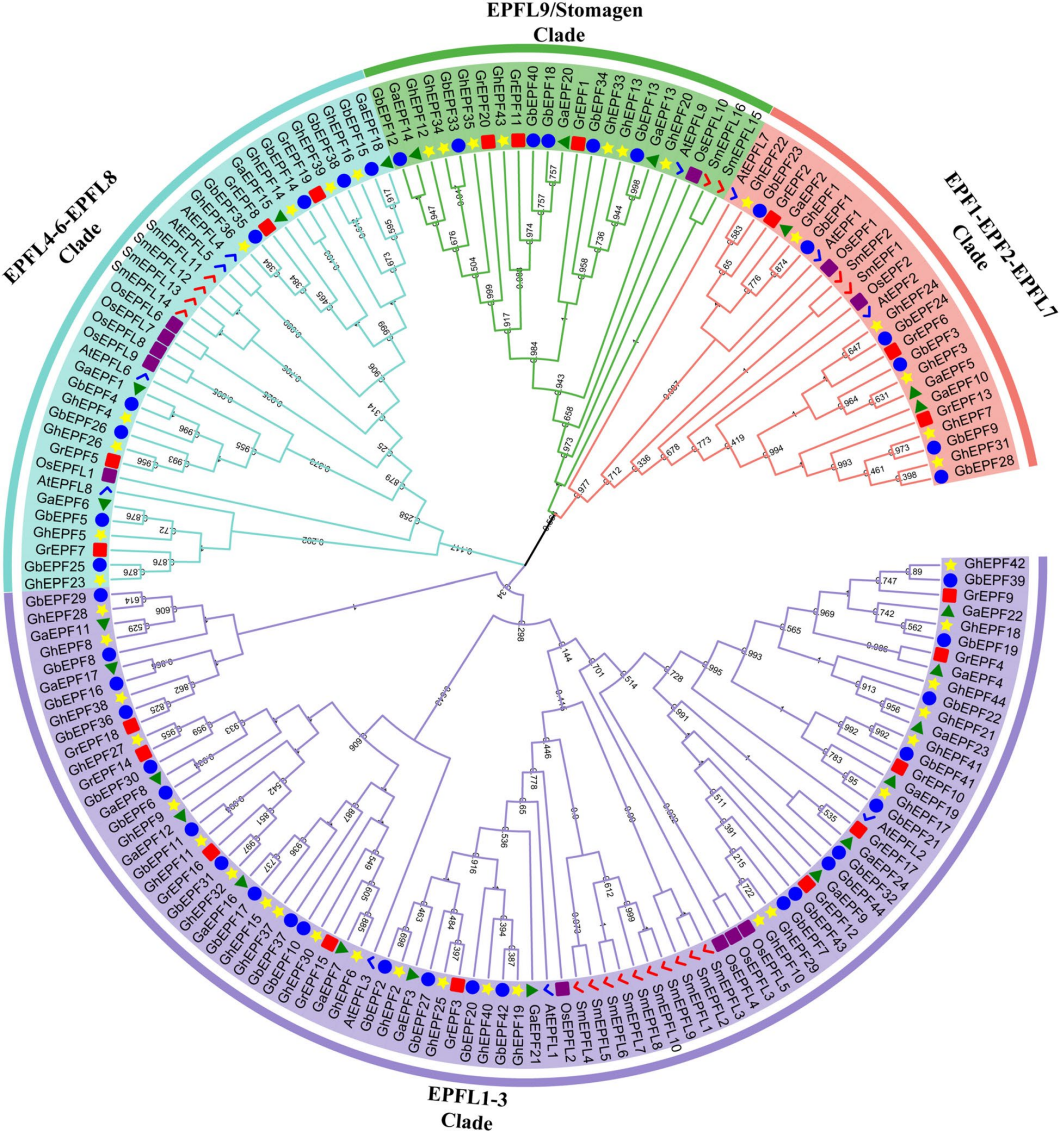
Phylogenetic analysis of EPF/EPFL proteins from *Gossypium*, *Arabidopsis thaliana*, Oryza sativa, and *Selaginella moellendorffii*. The blue checkmark presents the AtEPF/EPFL proteins, and the purple square presents the OsEPF/EPFL proteins. The red check mark presents the SmEPF/EPFL proteins, and the green triangle presents the GaEPF/EPFL proteins. The red square presents the GrEPF/EPFL proteins, and the yellow star and blue circle present the GhEPF/EPFL and GbEPF/EPFL proteins, respectively

The amino acid alignment of EPF/EPFLs were firstly conducted among the four cotton species, *A. thaliana, O. sativa, and S. moellendorffii* (Additional file 1: Figure S1), and the results showed the 6-cysteine residues conservatively located in the C-terminal mature peptide region for the both two clades. There were another 2-cysteine residues conserved in the loop region of EPF1-EPF2-EPFL7 clade, implying their diversity with EPFL9/Stomagen clade. Subsequently, 11 AtEPF/EPFL proteins, 13 OsEPF/EPFL proteins, and 17 SmEPF/EPFL proteins were utilized in this study to construct evolutionary tree together with 132 cotton EPF/EPFL proteins, resulting in four clades by virtue of neighbor-joining method (Fig. 1). The largest number of cotton EPF/EPFL proteins (71 ones, 53.8% of the total 132 ones) were classified into one clade with AtEPFL1-3 of the plant model species, therefore this clade was named as EPFL1-3. The most EPF/EPFL proteins of *A. thaliana*, namely as AtEPFL4, AtEPFL5, AtEPFL6, and AtEPFL8, and the second largest number of cotton EPF/EPFL proteins (24 ones, 18.2%) were divided into the same clade named as EPFL4-6-EPFL8. Eighteen EPF/EPFL proteins in cotton (13.6%) and 3 AtEPF/EPFL proteins, namely as AtEPF1, AtEPF2, and AtEPFL7, were classified into EPF1-EPFL2-EPFL7 clade, and 19 cotton EPF/EPFL proteins (14.4%) and AtEPFL9/Stomagen were divided into EPFL9/Stomagen clade.

## Chromosomal location and gene duplication of cotton EPF/EPFLs

**Fig. 2:**
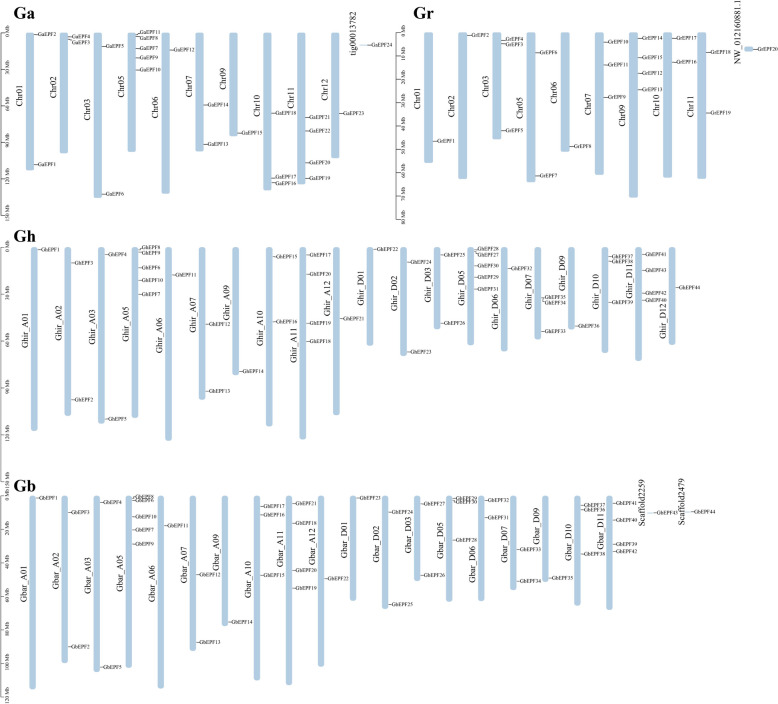
Chromosomal location of cotton EPF/EPFL genes

As shown in Fig. 2, 24 *GaEPF/EPFL* genes were unevenly distributed in 10 chromosomes and 1 scaffold except for 4th, 8th, and 13th chromosome in A2 genome, of which the numerous members were found in 5th (5 *GaEPF/EPFL* genes) and 11th (4 *GaEPF/EPFL* genes) chromosome. The similar results of 20 *GrEPF/EPFL* genes also occurred in D5 gnome, and there was no *GrEPF/EPFL* genes in 12th chromosome besides 4th, 8th, and 13th chromosome, while the different fact was that 9th chromosom chromosome harbored the most number (4 *GrEPF/EPFL* genes). As for the two AADD genomes, we noticed the same *EPF/EPFL* members and chromosome distributions between *G. hirsutum* and *G. barbadense*. The main difference was found that AD1 genome had two more *GhEPF/EPFL* genes separately located in D05 and D07 chromosomes, while AD2 genome had two more *GbEPF/EPFL* genes separately located in A10 and D06 chromosomes. With the comparison between the allotetraploid and diploid genomes, there were 21 *GhEPF/EPFL* genes and 22 *GbEPF/EPFL* genes in A subgenomes, and the numbers were less than 24 *GaEPF/EPFL* genes. On the contrary, 24 *GhEPF/EPFL* genes and 22 *GbEPF/EPFL* genes were found in D subgenomes that were more than 20 *GrEPF/EPFL* genes, despite their total number of *EPF/EPFL* genes were the same as 44. Besides, the distributed chromosomes between A2 and AD1 or AD2 were one-to-one corresponding, and we found one *GhEPF/EPFL* gene located in D12 chromosome in AD1 genome while not in D5 and AD2 genomes. These similarities and differences derived from the above results indicated *EPF/EPFL* gene family showed the conservatism and variability during the long-term evolutionary process.

## Gene structure and conserved motif prediction of cotton EPF/EPFLs

**Fig. 3:**
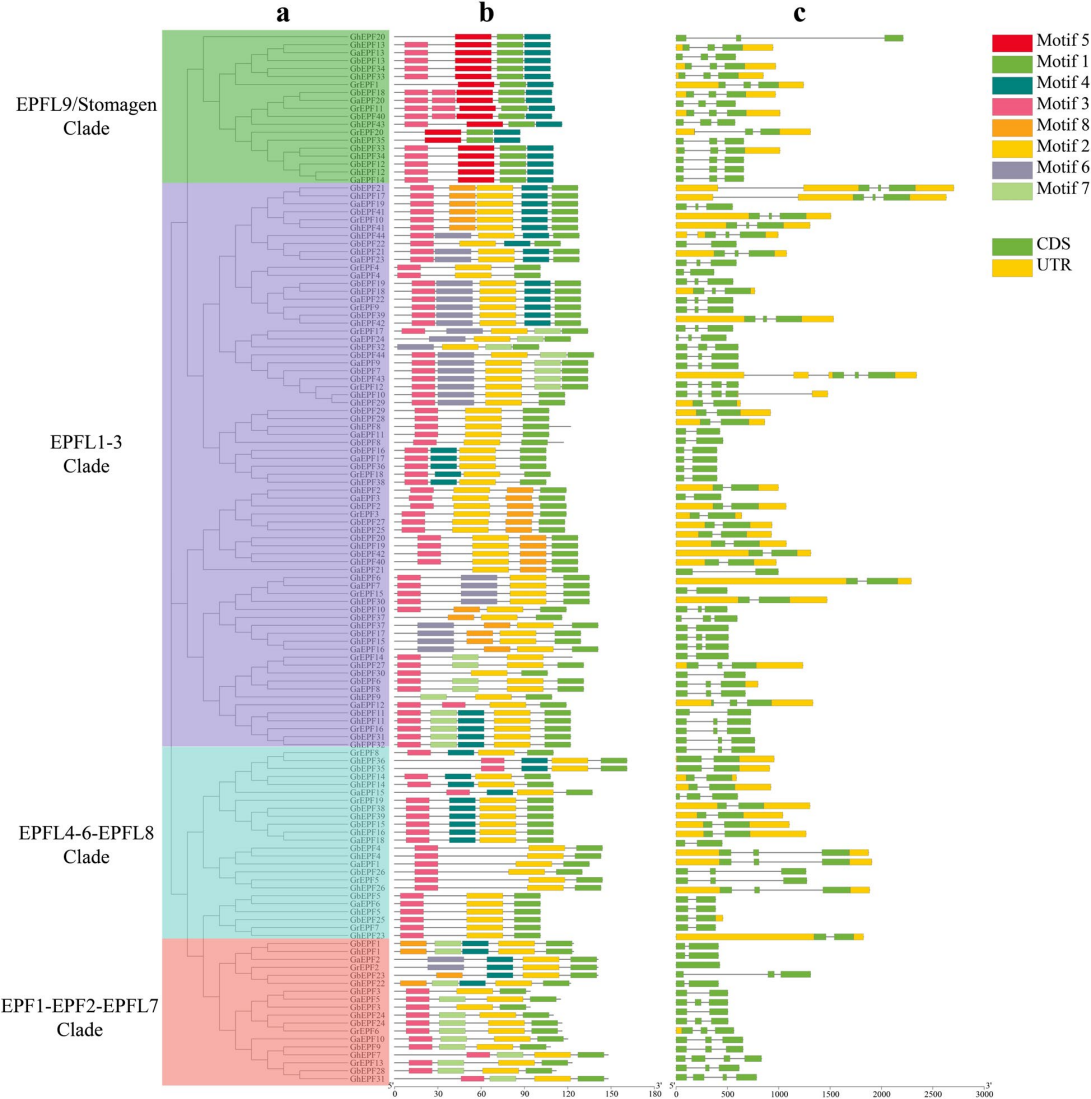
Gene structure and conserved motif identification of cotton EPF/EPFLs. a represents the evolutionary relationships of cotton EPF/EPFL genes, and b and c separately represent the conserved motifs and gene structures of cotton EPF/EPFL genes

The evolutionary relationships were also investigated among the cotton EPF/EPFLs based on their protein sequences, and the clustering result of 4 clades (Fig. 3a) maintained the consistency with the increased *Arabidopsis* EPF/EPFL proteins (Fig. 1). The prediction analysis of conserved motifs on the cotton EPF/EPFL proteins (Fig. 3b) indicated that a total of 8 conserved motifs were identified and named as Motif 1 to Motif 8 in turn (Additional file 2: Figure S1). The number of conserved motifs of cotton EPF/EPFLs ranged from 3 to 5, of which the most ones had 4 conserved motifs. Only Motif 1 was commonly observed in all the EPF/EPFL proteins, therefore it was deemed as the most conserved motif with the second lowest E-value. Motif 2 with the minimum E-value and the longest width was also commonly identified among the EPF/EPFL proteins of EPFL1-3, EPF1-EPF2-EPFL7, and EPFL4-6-EPFL8 clades, and Motif 3 with the third lowest E-value and the shortest width was also found among the PF/EPFL proteins of EPFL1-3, EPFL4-6-EPFL8, and EPFL9/Stomagen clades. Besides, we noticed that Motif 5 uniquely while commonly appeared in EPFL9/Stomagen clade, implying its potential significance.

The results of gene structure of cotton *EPF/EPFL* genes showed that the exon number of cotton *EPFL/EPFL* genes ranged from 1 to 4 (Fig. 3c). The main gene structure of cotton EPF/EPFL genes contained 3 exons and 2 introns (60/132), followed by the gene structures with 2 exons and 1 intron, with 4 exons and 3 introns, and with 1 exon and 0 intron.

## Collinearity analysis of EPF/EPFL genes in cotton

**Fig. 4:**
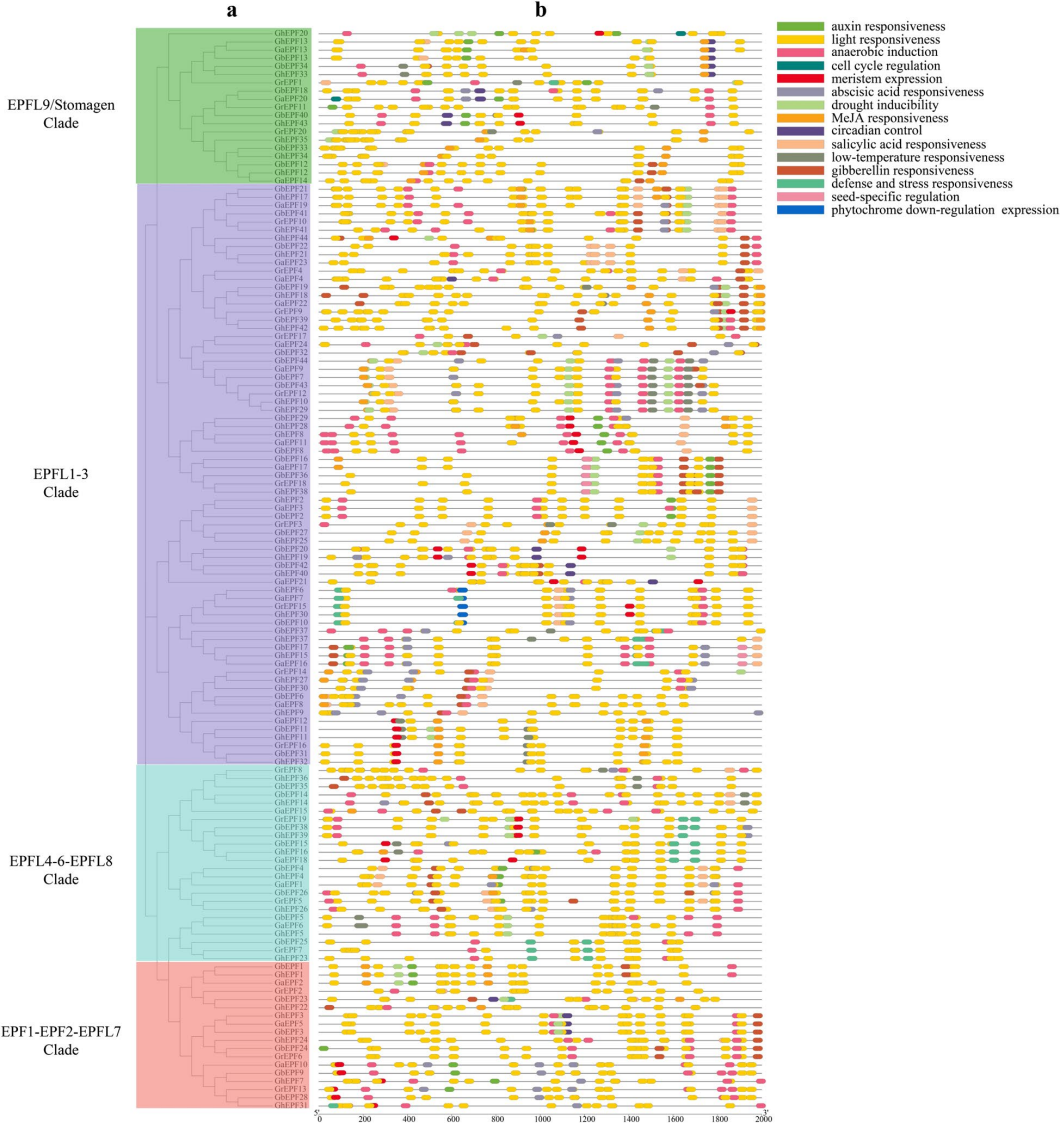
Collinearity events of duplication gene pairs of *EPF/EPFL* genes in four cotton species. The different color rectangles represented the chromosomes derived from the different cotton species, and the different color lines represented the collinearity relationships between and among the different cotton species

Gene duplication has been deemed as the main force to extend the number of gene family, which was generally consisted with tandem duplication, fragment duplication, and whole genome duplication [60]. We noticed that the EPF/EPFL numbers (44 ones) in allotetraploid cotton species were the sum numbers of *GaEPF/EPFL* (24 ones) and *GrEPF/EPFL* (20 ones) genes in the two diploid cotton species. The non-strict duplication phenomenon not only indicated the expansion of EPF/EPFL genes occurred in cotton polyploidy, but also implied the preference in different sub-genome during the evolution. The intra-specific and inter-specific collinearity was analyzed in the four cotton species (Fig. 4), and a total of 740 pairs of duplication genes were separately observed in the pairwise comparisons of Ga-Ga (18), Ga-Gr (47), Ga-Gh (99), Ga-Gb (90), Gr-Gr (12), Gr-Gh (87), Gr-Gb (75), Gh-Gh (78), Gh-Gb (175), and Gb-Gb (59). These results indicated that gene duplication might make difference in the expansion of *EPF/EPFL* gene family in cotton.

## Analysis of *cis*-regulatory elements in promoter regions of cotton *EPF/EPFL* genes

**Fig. 5:**
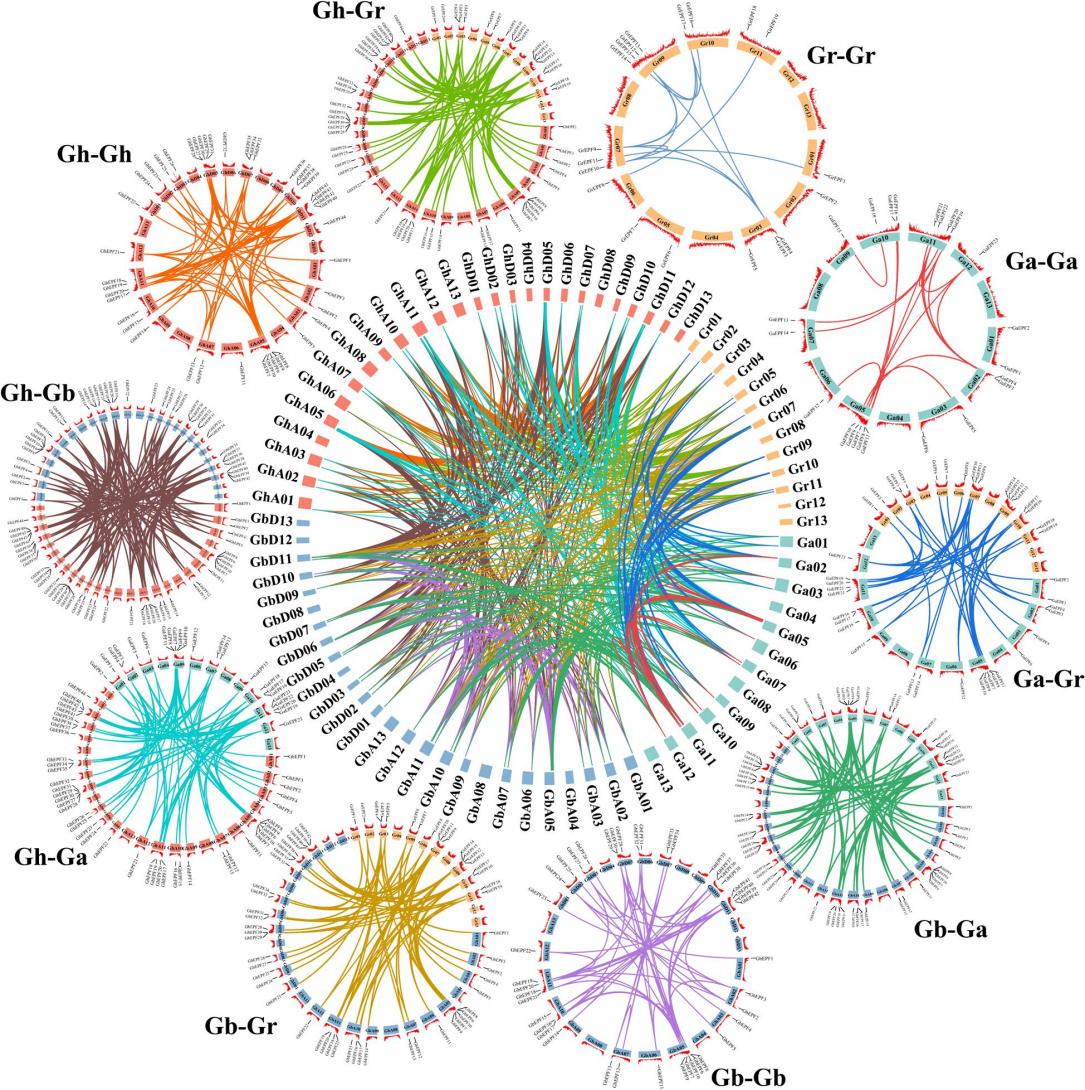
Cis-regulatory elements in the promoter regions of cotton EPF/EPFL genes

Fifteen kinds of cis-regulatory elements were totally identified in this study, and could be divided into three major categories that separately participate in the biological processes of development and growth, plant hormone response, and abiotic stress response (Fig. 5). The cis-regulatory elements relevant to plant development and growth included anaerobic induction, cell cycle regulation, meristem expression, circadian control, seed-specific regulation, and phytochrome down-regulation expression, and those hormone-related elements contained auxin responsiveness, abscisic acid responsiveness, MeJA responsiveness, salicylic acid responsiveness, and gibberellin responsiveness. Light responsiveness, drought inducibility, low-temperature responsiveness, and defense and stress responsiveness were the cis-regulatory elements of abiotic stress response. Overall, the largest number of cis-regulatory element located in cotton EPF/EPFL genes was light responsiveness, and another two enriched cis-regulatory elements were drought inducibility and MeJA responsiveness. These useful information will be conducive to excavating and verifying the possible function of cotton EPF/EPFLs in biological processes.

## Expression-pattern investigation and potential functions verified by qRT-PCR experiment

**Fig. 6:**
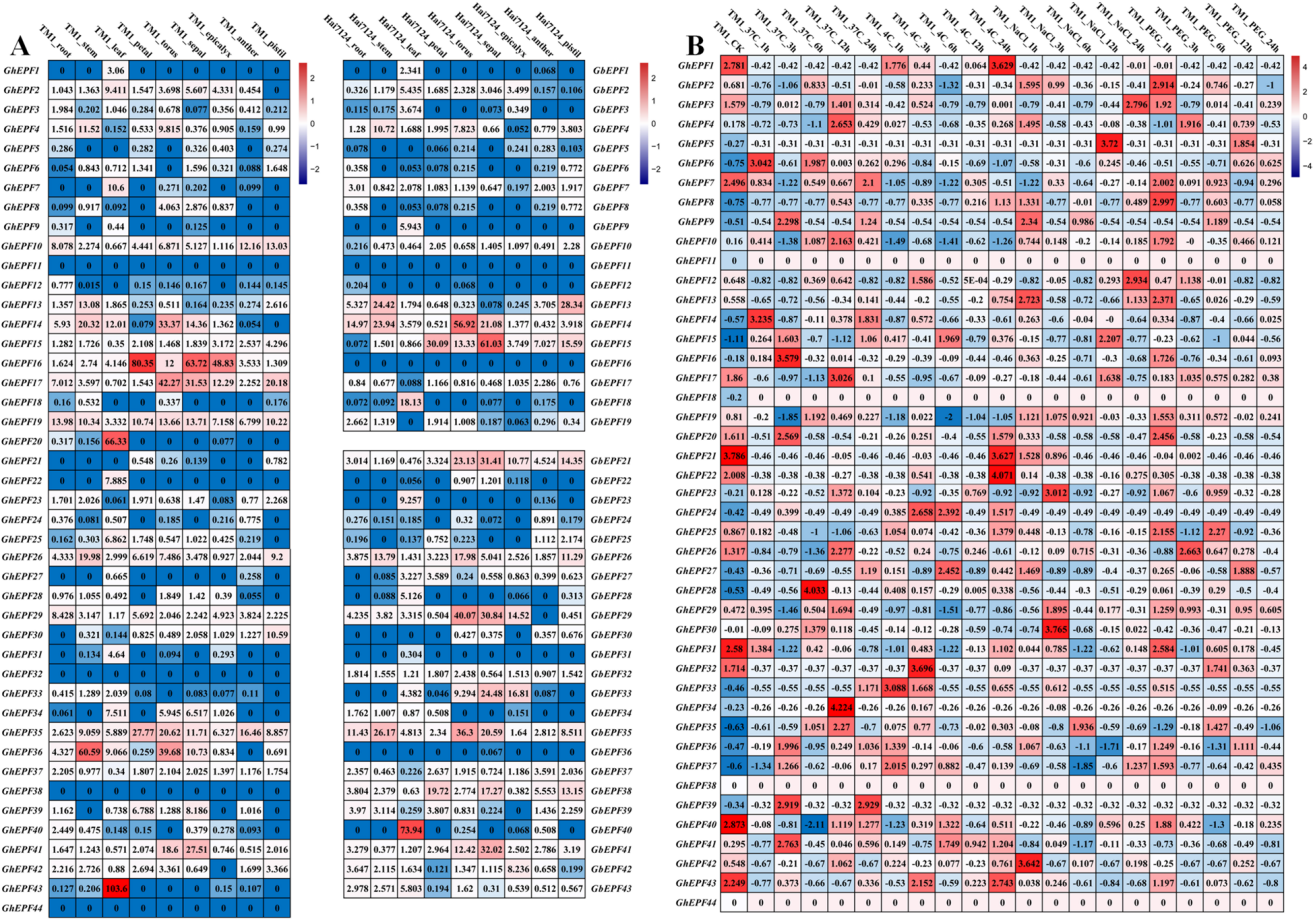
The tissue-specific expression and expressed patterns responding to abiotic stresses. A presented the analyses of specific expression of *GhEPF/EPFL* and *GbEPF/EPFL* genes in 9 tissues, and B presented the expression patterns of *GhEPF/EPFL* genes in response to high-temperature (37℃ treatment), low-temperature (4℃ treatment), salt stress (NaCl treatment), and drought stress (PEG treatment) at 0 h, 1 h, 3 h, 6 h, 12 h, and 24 h, respectively

Most of *EPF/EPFL* genes in the allotetraploid cotton species presented the relatively low expression levels (fragments per kilobase of exon model million mapped fragments, FPKM value < 10) in the selected tissues or organs (Fig. 6A). In TM-1, the highest expression levels were observed on *GhEPF19* in root, *GhEPF36* in stem, *GhEPF43* in leaf, *GhEPF16* in petal, *GhEPF17* in sepal, *GhEPF16* in epicalyx, *GhEPF33* in anther, and *GhEPF17* in pistil. While there were no homologous genes of *GbEPF20* and *GbEPF44* referred to RNA-seq data of Hai7124 tissues, and only *GbEPF14* in root, *GhEPF35* in stem, *GhEPF40* in leaf, *GhEPF15* in petal, *GhEPF14* in sepal, *GhEPF15* in epicalyx, *GhEPF33* in anther, and *GhEPF13* in pistil were highly expressed. In general, most of cotton *EPF/EPFL* genes were expressed at low levels in the tissues or organs.

The normalized data indicated that the majority of *GhEPF/EPFL* genes showed down-regulated expression patterns responding to the abiotic stresses (Fig. 6B), besides 4 *EPF/EPFL* genes (*GhEPF11*, *GhEPF18*, *GhEPF38*, and *GhEPF44*) were completely not expressed among the different treatments. Interestingly, nearly half of *GhEPF/EPFL* genes presented up-regulated expression pattern during the high-temperature stress (from 0 to 24 h), when *GhEPF6*, *GhEPF14-16*, *GhEPF20*, *GhEPF31*, *GhEPF36*, and *GhEPF41* showed down-regulated expression pattern. The *GhEPF/EPFL* genes were mainly down-regulated under low-temperature stress, of which *GhEPF1*, *GhEPF22*, *GhEPF33*, *GhEPF37*, and *GhEPF43* showed firstly down-regulated and then up-regulated expression pattern, while *GhEPF15*, *GhEPF27*, *GhEPF40*, and *GhEPF41* presented firstly up-regulated and then down-regulated expression pattern. Similarly as in response to high-temperature stress, we found approximately 50% of *GhEPF/EPFL* genes showed continuously down-regulated expression pattern against NaCl treatment, and *GhEPF3*, *GhEPF12*, *GhEPF22*, and *GhEPF37* presented the gradually up-regulated meanwhile *GhEPF26* and *GhEPF35* showed the firstly up-regulated while then down-regulated expression patterns. With regard to PEG treatment, most of *GhEPF/EPFL* genes presented continuously down-regulated expression pattern, while only a small number of *EPF/EPFL* genes showed the gradually up-regulated or firstly up-regulated and then down-regulated expression patterns, of which the former included *GhEPF5*, *GhEPF6*, and *GhEPF27*, and the latter contained *GhEPF9*, *GhEPF25*, *GhEPF32*, and *GhEPF35*. By and large, dramatically expressed differences were observed in the cotton *EPF/EPFL* genes responding to high/low temperature and salt or drought stress, implying their potential functions in the multiple adversity stresses.

**Fig. 7:**
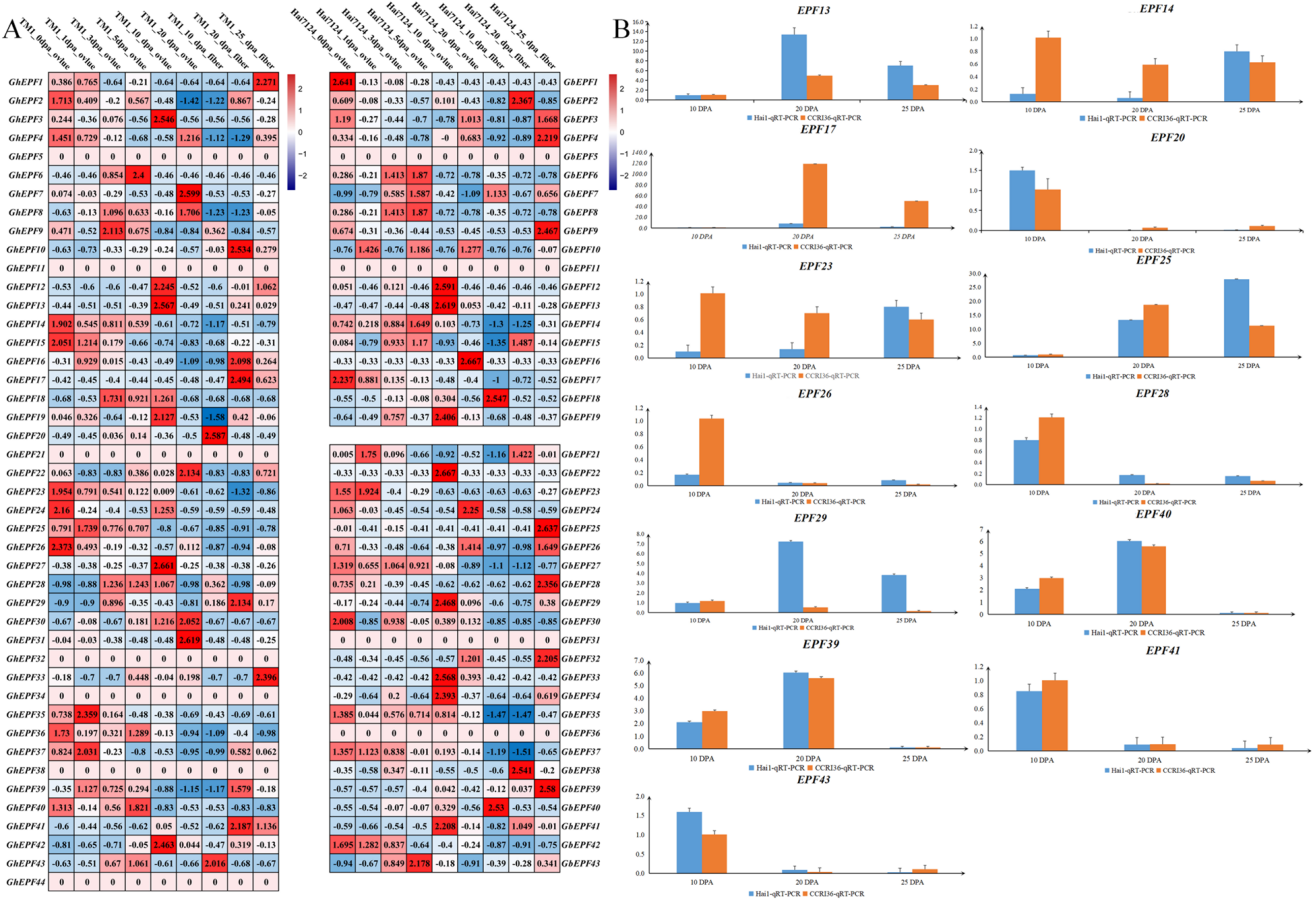
The expression patterns and qRT-PCR verification of cotton *EPF/EPFL* genes during fiber development. A presented the analyses of expression patterns of *GhEPF/EPFL* and *GbEPF/EPFL* genes on the developing ovule (0, 1, 3, 5, 10, and 20 DPA) and fiber (10, 20, and 25 DPA), and B presented the qRT-PCR verification of 15 highly expressed *GhEPF/EPFL* genes on the high-yield and wide-adaptability CCRI36 and superior fiber-quality and high VW-resistance Hai1 at 10, 20, and 25 DPA, respectively

The results of expression patterns during development of ovule and fiber (Fig. 7A) showed thata total of 7 *EPF/EPFL* genes showed no differences among the developing ovules or fibers in TM-1, namely *GhEPF*5, *GhEPF11*, *GhEPF21*, *GhEPF32*, *GhEPF34*, *GhEPF38*, and *GhEPF44*, while the similar phenomenon was also observed in *GbEPF5*, *GbEPF11*, *GbEPF31*, and *GbEPF36* of Hai7124 ovules and fibers.

During the initial period (0 to 3 DPA) of ovule development, 8 of 44 *GhEPF/EPFL* genes showed the highest up-regulated fold changes at 0DAP, namely as *GhEPF2*, *GhEPF4*, *GhEPF14*, *GhEPF15*, *GhEPF23*, *GhEPF24*, *GhEPF26*, and *GhEPF36,* which were observed at 1 DPA as *GhEPF25*, *GhEPF35*, *GhEPF37*, and *GhEPF39,* while at 3 DPA as *GhEPF9*, *GhEPF18*, and *GhEPF28*. As for the elongation period (5 to 20 DPA) of ovule development, we noticed that *GhEPF6*, *GhEPF36*, *GhEPF40*, and *GhEPF43* at 5 DPA, *GhEPF3*, *GhEPF12*, *GhEPF13*, *GhEPF18*, *GhEPF19*, *GhEPF24*, *GhEPF27*, and *GhEPF42* at 10 DPA, and *GhEPF7*, *GhEPF8*, *GhEPF22*, *GhEPF30*, and *GhEPF31* at 20 DPA, showed the highest up-regulated fold changes. During the elongation and secondary wall thickening periods (10 to 25 DPA) of fiber development, *GhEPF20* and *GhEPF43* at 10 DPA, *GhEPF10*, *GhEPF16*, *GhEPF17*, *GhEPF29*, *GhEPF39*, and *GhEPF41* at 20 DPA, and *GhEPF1*, *GhEPF33*, and *GhEPF41* at 25 DPA harbored the highest up-regulated fold changes. Similarly in the ovule and fiber development of Hai7124, we identified the highest up-regulated fold changes as *GbEPF1*, G*bEPF17*, *GbEPF30*, and *GbEPF42* in the ovule at 0 DPA, *GbEPF10* and G*bEPF21* in the ovule at 1 DPA, *GbEPF6-8*, *GbEPF14*, and *GbEPF43* in the ovule at 5 DPA, *GbEPF12*, *GbEPF13*, *GbEPF19*, *GbEPF21*, *GbEPF29*, *GbEPF33*, *GbEPF34*, and *GbEPF41* in the ovule at 10 DPA, *GbEPF16* and *GbEPF24* in the ovule at 20 DPA, *GbEPF18* and *GbEPF40* in the fiber at 10 DPA, *GbEPF2*, *GbEPF15*, and *GbEPF38* in the fiber at 20 DPA, and *GbEPF3*, *GbEPF4*, *GbEPF9*, *GbEPF25*, *GbEPF26*, *GbEPF28*, *GbEPF32*, and *GbEPF39* in the fiber at 25 DPA. These data indicated that cotton *EPF/EPFL* genes might play important roles affecting the development and growth of ovules and fibers.

After conducting the relative quantization on the 13 high-expressed *GhEPF* genes during the key development periods of cotton fiber (10, 20, and 25 DPA), the largest number of *GhEPF* genes showed the dramatic changes on the expression levels either between the two different varieties or among the three developing periods, while little changes were mainly observed in *GhEPF20*, *GhEPF26*, and *GhEPF43* between CCRI36 and Hai1 at 20 and 25 DPA. As for the fiber-elongation period (10 DPA), we noticed *GhEPF10*, *GhEPF26*, and *GhEPF43* presented higher expression levels in Hai1 than those in CCRI36. During the secondary-wall thickening periods, *GhEPF13*, *GhEPF28*, *GhEPF29*, *GhEPF39*, *GhEPF40*, and *GhEPF43* were more highly expressed in Hai1 than those in CCRI36 at 20 DPA, and *GhEPF13*, *GhEPF14*, *GhEPF23*, *GhEPF25*, *GhEPF26*, *GhEPF28*, and *GhEPF29* showed higher expression levels in Hai1 than those in CCRI36 at 25 DPA. To sum up, despite there were no *GhEPF* genes harboring the steadily higher expression levels in Hai1 under the key developmental periods of fiber elongation and secondary-wall thickening periods, *GhEPF26* and *GhEPF43* were separately observed to highly expressed in sea island cotton than those in upland cotton between 10 DAP and 25 DPA, and between 10 and 20 DPA, while *GhEPF13*, *GhEPF28*, and *GhEPF29* were found with higher expression levels in sea island cotton than those in upland cotton between 20 and 25 DPA. These highly expressed *GhEPF* genes might be the candidate genes determining why the sea island cotton had the superior fiber quality than upland cotton, which definitely require the further experiments of genetic transformation to verify their potential functions in fiber development.

## Analyses of protein–protein interaction and functional enrichment of *GhEPF/EPFL* genes

**Fig. 8:**
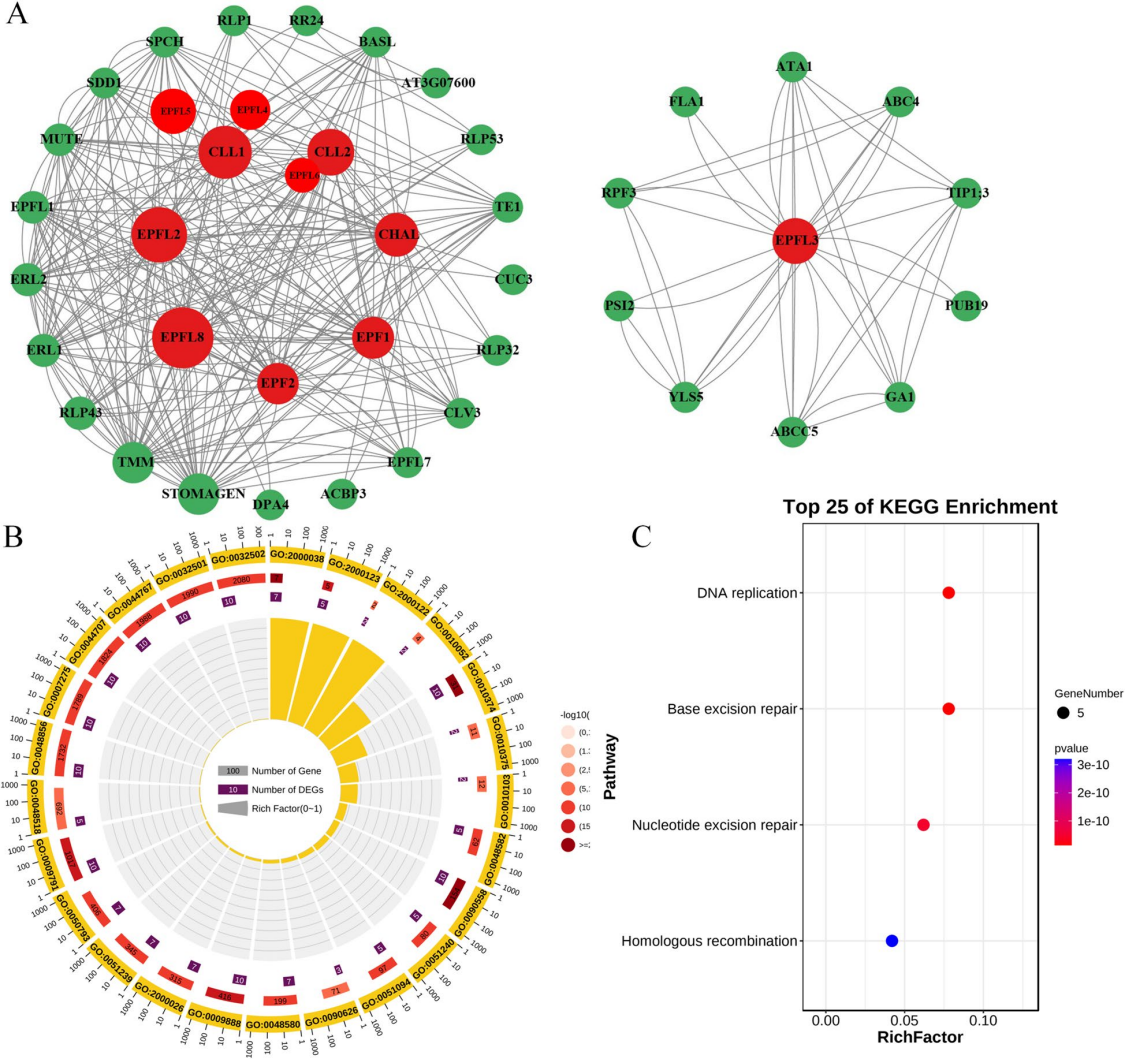
The protein–protein interaction and functional enrichment of *GhEPF/EPFL* genes. A presented the analysis of protein–protein interaction of *GhEPF/EPFL* genes based on the String database, and B and C separately presented the enriched GO categories and KEGG pathways of *GhEPF/EPFL* genes

For screening the candidate *EPF/EPFL* genes that could be utilized to cultivate the high-yield, superior fiber-quality, and multiple-resistance varieties in cotton by over-expression and gene-editing technology, the analyses of protein–protein interaction and functional enrichment were conducted on the cotton *EPF/EPFL* genes, respectively. Due to the fact that there was no uploaded protein data of cotton species in the STRING database (http://string-db.org/), homologous alignment was firstly carried out between the EPF/EPFL proteins of *A. thaliana* and *G. hirsutum*, which were subsequently utilized to draw the interaction networks together with their interacting proteins (Fig. 8A). The results indicated that the core interaction networks concentrated on the eight EPF/EPFL proteins of *A. thaliana*, namely EPFL8 (homologous proteins as GhEPF8, GhEPF28, and GhEPF38), EPFL2 (homologous proteins as GhEPF10, GhEPF17, GhEPF18, GhEPF21, GhEPF29, GhEPF41, and GhEPF42), EPFL5 (homologous protein as GhEPF39), EPFL4 (homologous proteins as GhEPF5, GhEPF14, GhEPF16, GhEPF23, and GhEPF36), EPFL6 (homologous proteins as GhEPF4 and GhEPF26), EPF1 (homologous protein as GhEPF1), EPF2 (homologous proteins as GhEPF3, GhEPF7, GhEPF24, and GhEPF31), and EPFL3(homologous proteins as GhEPF6, GhEPF9, GhEPF11, GhEPF15, GhEPF27, GhEPF30, GhEPF32, and GhEPF37) ordered by the interacting extents. In order to further investigate the potential functions of these GhEPF/EPFL proteins, enrichment analyses of GO and KEGG pathways were separately accomplished as shown in Fig. 8B and 8C. As for the GO categories, the most significant enriched terms were stomatal complex development (GO:0010374), regulation of stomatal complex development (GO:2,000,038), and plant epidermis development (GO:0090558) in biological processes, membrane (GO:0016020), integral component of membrane (GO:0016021), and membrane part (GO:0044425) in cellular component, and hydrolase activity (GO:0016787), hydrolase activity (GO:0016787), and nucleotide binding (GO:0000166) in molecular function. Among the annotated *EPF/EPFL* genes in GO terms, *GhEPF1*, *GhEPF7*, *GhEPF13*, *GhEPF20*, *GhEPF22*, *GhEPF24*, *GhEPF31*, *GhEPF33*, *GhEPF34*, and *GhEPF43* were the most enriched ones that participate in the top 10 biological processes. While, only DNA replication (ko03030), base excision repair (ko03410), nucleotide excision repair (ko03420), and nucleotide excision repair (ko03440) were the enriched KEGG pathways by the same 5 *EPF/EPFL* genes, namely *GhEPF1*, *GhEPF7*, *GhEPF22*, *GhEPF24*, and *GhEPF31*. The comprehensive analyses of protein–protein interaction and functional enrichment on the *GhEPF/EPFL* genes indicated the two comment enriched genes were found namely as *GhEPF1* and *GhEPF7*, which might deserve more attentions for further functional verification and interacting-mechanism research.

## Discussion

Cotton is one of the most widely cultivated cash crops all around the world, whose natural fiber, edible oil, vegetable protein, and other products have greatly contributed to the national economy and social development [61]. However, the cotton production is suffering the increasingly serious disputes on arable lands between grain and cotton. The deteriorating ecological environment by salinization phenomenon and extreme weather (high/low temperature or flood and drought), the ever-expanding diseases (*Verticillium* wilt and *Fusarium* wilt) and pets (cotton bollworms and aphids), while the growing population of human beings and the rising living standards and consumption levels, resulting in that how to simultaneously improve the fiber yield and quality so as to cultivate and promote new varieties with high production, superior quality, and multiple resistance has become a different issue to be solved urgently at present [62–64]. Therefore, it is of necessity and significance to concentrate on the functional genes relevant to fiber development and growth since the specific biological process could determine the final fiber yield and quality. Coincidentally, *EPF/EPFL* gene family was annotated as the epidermal patterning factor [65], and cotton fiber was a single-cell seed trichome differentiated from the epidermal cell of ovule [66], implying the close relationships between the two above-mentioned ones. Despite there was few reports on *EPF/EPFL* genes in cotton, *EPF/EPFL* gene family has been confirmed in multiple plant species to participate in plant development and growth, especially as the morphogenesis processes of stoma, awn, stamen, and fruit skin [14–31]. Bases on these facts, the four representative cotton species were chosen in this study to perform the genome-wide identification and analyses of chromosome location, gene structure, conserved motifs, and collinearity relationship on the *EPF/EPFL* gene family, meanwhile the published RNA-seq data were also utilized to investigate their specific-tissue expression and responding patterns to developing processes of ovule and fiber and to abiotic stresses. Additionally, protein database and on-line tools were used for analyzing the interacting network and functional enrichment of cotton EPF/EPFL proteins, and these results help us screen some candidate genes for further transgenic verification and provide abundant genetic resources for molecular breeding in cotton production.

In this study, a total of 132 *EPF/EPFL* genes were identified from two diploid and two tetraploid cotton species, which were composed of 20 *G. raimondii* (D5), 24 *G. arboreum* (A2), 44 *G. hirsutum* (AD1), and 44 *G. barbadense* (AD2), respectively. Compared with 11 *EPF/EPFL* members in *A. thaliana* and apple while 12 *EPF/EPFL* members in rice [11–13], the same diploid cotton species had more numbers of *EPF/EPFL* genes, which might be relevant to their differences on genome complexity and evolutionary origins while not depend on the more or less of chromosome numbers and belonging phyla of herbaceous or ligneous plants. Interestingly, the *GhEPF/EPFL* or *GbEPF/EPFL* number was the sum number of *GrEPF/EPFL* and *GaEPF/EPFL* genes, while the tetraploid ones were not strictly twice of the diploid ones, which was proved by the non-absolute one to one correspondences of chromosome locations of EPF/EPFL gene between A-subgenome or D-subgeome of allotetraploid cotton species and A2 or D5 of diploid cotton species (Fig. 2). The fact of the incomplete equilibrium in cotton *EPF/EPFL* gene family indicated the conservatism and variability during the natural hybridization and chromosome reduplication. In the meantime, the on-line tools were utilized to predict the physical and chemical characteristics, and either amino acid length and molecular weight or isoelectir point and instability index widely ranged among all the cotton *EPF/EPFL* proteins. The largest number of cotton *EPF/EPFL* gene family were predicted to be located in chloroplast and extracellular, of which the distribution in former location might be related with ROS (reactive oxygen species) concentrations caused by some over-expressed *EPF/EPFL* genes against adversity stresses, such as *MdEPF2* [13], while the latter location indicated these secretory polypeptides might combine with receptors on the cell membrane to transfer the extracellular signals and to play important roles in the process of signaling between cells [67, 68].

A total of 173 EPF/EPFL proteins were used to construct the phylogenetic tree, including 11 AtEPF/EPFL, 13 OsEPF/EPFL, 17 SmEPF/EPFL, and 132 cotton EPF/EPFL proteins, which were divided into four clades (Fig. 1). Each clade was composed of four kinds of EPF/EPFLs derived from four cotton species, and was separately named as EPF1-EPF2-EPFL7, EPFL1-3, EPFL4-6-EPFL8, and EPFL9/Stomagen according to the included AtEPF/EPFLs, which was consistent with the previous studies [11–13]. After removing the AtEPF/EPFL proteins, all the cotton EPF/EPFLs were also subjected to investigate their evolutionary relationships (Fig. 3a), and the similar clustering results in gene structure and conserved motif confirmed the phylogenetic categories. Besides, we noticed that the number of conserved motifs ranged from 3 to 5 in the cotton EPF/EPFL proteins, while only Motif 1 was commonly found in all the EPF/EPFLs (Fig. 3b). The numbers of exon and intron of cotton *EPF/EPFL* genes ranged from 1 to 4 and 0 to 3, respectively, of which the main gene structure was composed of 3 exons and 2 introns (Fig. 3c). Despite more *EPF/EPFL* genes were observed in cotton species, the evolutionary classification, conserved motif, and exon–intron structure were generally accorded with those in *A. thaliana*, *O. sativa* and *M. domestica* [11–13], indicating the high conservation during the natural evolution and species expansion.

The uneven distribution of cotton EPF/EPFL genes in the chromosomes between the tetraploid subgenome and the diploid genome promoted us to perform the collineary analysis for estimating the possible reasons. As we all know, gene duplication has been proved to provide the main contribution to the extension of gene family, generally including tandem duplication, fragment duplication, and whole genome duplication [39]. The intraspecific and interspecific results of collineary relationships among the four cotton species showed the total duplication genes were 740 pairs (Fig. 4), which provided strong evidences that gene duplication could make difference in expanding *EPF/EPFL* genes like other families in cotton [69, 70]. The increased size and complexity of plant genome were accomplished by the events of chromosome doubling and gene duplication, which also made it harder to accurately assembly and obtain the whole genome information even though combing the second-generation and third-generation sequencing technologies with High-through chromosome conformation capture (Hi-C) and Bionano optical maps [32, 38]. The inevitable mistakes and unassembled scaffolds in cotton genomes might also be cause for explaining the non-strict duplication phenomenon between the tetraploid and diploid species.

The gene functions were closely related with their coding regions, while their transcriptions did occur or not were up to the combination between the transcription factors and promoter regions [71]. Cis-regulatory elements of the cotton EPF/EPFL genes were analyzed to comprehend their potential functions in the multiple biological processes, and the 15 identified kinds of cis-regulatory elements were divided into three categories: plant development and growth, plant hormone response, and abiotic stresses (Fig. 5). The most researches of EPF/EPFL functions were concentrated on the stoma development [9, 10, 12] followed by the development of awn and stamen [18–20], which indicated the important roles in coordinating plant development and growth. What is more, the awn-related gene *OsEPFL2* was proved to promote the leaf margin morphogenesis by combing with ER family receptor-kinases meanwhile with adequate auxin supply in *A. thaliana* [20], and the over-expressed *MdEPF2* was found to balance the drought tolerance and waiter-use efficiency by responding ABA hormone [13], implying the close relationships between EPF/EPFL genes and plant hormone response. Apart from *MdEPF2*, another *EPF3* gene in *Populus deltoides* was ectopically expressed in *A. thaliana* to improve the drought tolerance by reducing the stomatal density [72], and these results illustrated that the cis-regulatory elements could guide us to explore the potential functions to affect the specific biological processes.

The specific-tissue expression analysis was performed on the 44 *GhEPF/EPFL* and 42 *GbEPF/EPFL* genes with the previous RNA-seq data of 9 tissues/organs in the two tetraploid cotton species namely as TM-1 and Hai7124 [32], and the results showed that the largest number of cotton *EPF/EPFL* genes were expressed at low levels in the tissues and organs belonging either to vegetative growth or to reproductive growth (Fig. 6A). Only some *EPF/EPFL* genes harboring highly expressed levels in leaf, stem, petal, torus, and sepal might participate in these morphogenesis, which accorded with the functions of *AbEPF/EPFL* genes that regulated the inflorescence development and stomatal density [73]. In consideration of the enormous influence on the cotton yield and fiber quality caused by the multiple adversity stresses and the close relationship between the cotton EPF/EPFL functions and the predicted cis-regulatory elements of abiotic-stress responsiveness, the transcriptome data of high temperature, low temperature, salt stress, and drought treatment conducted on TM-1 were utilized in this study to investigate the expression patterns in response to abiotic stresses, and the dramatic changes were observed among the *44 GhEPF/EPFL* genes (Fig. 6B). On the whole, there were 4 *EPF/EPFL* genes with completely not expressed levels among the different treatments, namely *GhEPF11*, *GhEPF18*, *GhEPF38*, and *GhEPF44*, while another 10 *EPF/EPFL* genes basically maintained the same down-regulated status (*GhEPF1*, *GhEPF5*, *GhEPF9*, *GhEPF21*, *GhEPF22*, *GhEPF24*, *GhEPF32*, *GhEPF33*, *GhEPF34*, and *GhEPF39*) except for the up-regulated expression levels at some specific point-in-time. Besides, nearly half of *GhEPF/EPFL* genes presented up-regulated expression pattern during the high-temperature stress (37℃ from 1 to 24 h), while *GhEPF6*, *GhEPF14-16*, *GhEPF20*, *GhEPF31*, *GhEPF36*, and *GhEPF41* showed down-regulated expression pattern. The existing research results indicated the high temperature could seriously affect the stomatal density [74], while *EPF1-2* and *EPFL9/Stomagen* were found to play important roles in control of lead stomatal density [11, 12]. After homologous alignment between the proteins of 44 GhEPF/EPFL and 11 AtEPF/EPFL, we found the only up-regulated *GhEPF20* and *GhEPF31* were separately annotated as *AtEPFL9* and *AtEPF2* that could be the candidate genes for further investigating whether to affect the leaf stomatal density in cotton. As for the low-temperature treatment (4℃) from 1 to 24 h, most of *GhEPF/EPFL* genes showed the down-regulated expression pattern, and *GhEPF1*, *GhEPF22*, *GhEPF33*, *GhEPF37*, and *GhEPF43* were firstly up-regulated and then down-regulated, while *GhEPF15*, *GhEPF27*, *GhEPF40*, and *GhEPF41* presented firstly up-regulated and then down-regulated expression pattern. *EPFL6* was previously proved to come into play in the development of stamen and pistile under cool temperature [75], and its homologous genes namely as *GhEPF4* and *GhEPF26* showed the down-regulated expression pattern, implying the negative regulatory in cotton responding to low-temperature stress. With regard to salt stress, approximately 50% of *GhEPF/EPFL* genes showed continuously down-regulated expression pattern, and the pattern of gradually up-regulated expression was observed in *GhEPF3*, *GhEPF12*, *GhEPF22*, and *GhEPF37* meanwhile that of firstly up-regulated and then down-regulated expression was found in *GhEPF26* and *GhEPF35*. However, there was few reports directly pointing out the close relationships between *EPF/EPFL* genes and salt-stress responsiveness, leaving some blank space to be explored in the future. The largest number of *GhEPF/EPFL* genes showed the continuously down-regulated expression patterns during the drought stress periods (from 1 to 24 h), when *GhEPF5*, *GhEPF6*, and *GhEPF27* with the gradually up-regulated pattern while *GhEPF9*, *GhEPF25*, *GhEPF32*, and *GhEPF35* harboring the pattern of firstly up-regulated and then down-regulated expression were observed. The over-expression of *MdEPF2* in tomato was found not only to participate in the stomatal development, but also to improve the drought resistance and water use efficiency (WUE), and its homologous genes namely as *GhEPF3*, *GhEPF7*, *GhEPF24*, and *GhEPF31* might function as the negative regulatory elements in cotton responding to drought stress, which will be further verified by the genetic transformation technology.

The public RNA-seq data conducted on the developing ovules and fibers were utilized to explore the relationship between *EPF/EPFL* genes and plant development, and more *EPF/EPFL* genes with dramatic differences that those under abiotic stresses were observed in the Fig. 7A. Compared with the expression patterns between ovule and fiber development, we noticed that more *EPF/EPFL* genes showed the down-regulated expression patterns in fiber development, while more patterns of firstly down-regulated and then up-regulated expression were observed in ovule development. These data indicated the cotton *EPF/EPFL* genes presented the more dramatic differences to affect the ovule development, which could determine the final fiber yield [76]. The various changes of *EPF/EPFL* genes during the development of fibers also implied their significant roles to control fiber quality, therefore qRT-PCR experiments were adopted to verify and screen the potential candidate genes in two tetraploid cotton varieties harboring the extreme difference between the cotton yield and fiber quality (Fig. 7B). Most of the selected 15 *GhEPF/EPFL* genes were differentially expressed either in the two materials or during the different periods of fiber development, of which *GhEPF10*, *GhEPF26*, and *GhEPF43* presented higher expression levels in Hai1 than those in CCRI36 during the fiber-elongation period (10 DPA). As for the period of secondary cell-wall thickening, *GhEPF13*, *GhEPF28*, *GhEPF29*, *GhEPF39*, *GhEPF40*, and *GhEPF43* were more highly expressed in Hai1 than those in CCRI36 at 20 DPA, while *GhEPF13*, *GhEPF14*, *GhEPF23*, *GhEPF25*, *GhEPF26*, *GhEPF28*, and *GhEPF29* showed higher expression levels in Hai1 than those in CCRI36 at 25 DPA. On one hand, these differentially expresses *GhEPF/EPFL* genes could make contributions to fiber development, and on the other hand, the common high-expression *GhEPF26* between 10 and 25 DPA, *GhEPF43* between 10 and 20 DPA, and *GhEPF13*, *GhEPF28*, and *GhEPF29* between 20 and 25 DPA in the superior fiber-quality cotton variety provided the abundant gene resource for further genetic transformation verification and molecular breeding in cotton production.

Protein interacting network, also namely as protein–protein interaction (PPI) analysis, is composed of individual proteins interacting with each other, afterwards to participate in the biological signal transmission, gene expression regulation, energy and material metabolism, and cell cycle control [77]. PPI analysis has been widely utilized in the gene family researches to construct the protein interacting network for screening the core candidate genes [78–80], and our results also identified the core proteins namely as EPFL8, EPFL2, EPFL5, EPFL4, EPFL6, EPF1, EPF2, and EPFL3 (Fig. 8A). However, more homologous proteins were found in cotton genomes, which required other database to perform conjoint analyses for narrowing the range of the key candidate genes. The enrichment analyses of GO and KEGG pathways were adopted to excavate the potential functional categories and significant signal transduction (Fig. 8B and 8C), of which the most significant enriched biological processes of GO terms were stomatal complex development (GO:0010374), regulation of stomatal complex development (GO:2,000,038), and plant epidermis development (GO:0090558), and DNA replication (ko03030), base excision repair (ko03410), nucleotide excision repair (ko03420), and nucleotide excision repair (ko03440) were identified as the enriched KEGG pathways. The close relationship of cotton *EPF/EPFL* genes with stoma development and epidermis development were consistent with the previous studies [11–13]. Having employing the comprehensive analyses of PPI, GO enrichment, and KEGG pathway on the *GhEPF/EPFL* genes, *GhEPF1* and *GhEPF7* were finally screened as the common enriched genes for further research by cultivating transgenetic varieties and investigating the interacting-mechanism among the signal pathways.

## Conclusion

Genome identification of *EPF/EPFL* gene family was performed on the four representative cotton species, and 132 cotton EPF/EPFL proteins were divided into 4 clades based on their evolutionary relationships. The comprehensive analyses of chromosome location, gene structure, conserved motifs, and collineary relationship indicated the conservatism and variability during the natural hybridization and chromosome reduplication, and expression patterns in response to multiple abiotic stresses and during the development of ovules and fibers demonstrated that cotton *EPF/EPFL* genes participate in coordinating the adversity responsiveness and plant development. Finally, the crucial candidate *EPF/EPFL* genes were comprehensively screened by protein interacting network and enrichment analyses of GO and KEGG pathway, and these results not only provided useful information for further functional verification, but also established a solid foundation for molecular breeding in cotton production.

### Supplementary Information

Additional file 1: Table S1. Primer information of 15 *GhEPF/EPFL* and *Ubiquitin7* for qRT-PCR experiments. Table S2. Identification and physiochemical characteristic analysis of cotton EPF/EPFL genes.

Additional file 2: Figure S1. The conserved peptides among the EPF/EPFL proteins. Figure S2. The information of 8 conserved motifs.

## Data Availability

All data generated or analyzed in this study are included in this published article.
