# Supplementary material for: Genome-wide analyses of member identification, expression pattern, and protein–protein interaction of EPF/EPFL gene family in Gossypium
Source: BMC Plant Biol. 2024 Jun 14;24:554. doi: 10.1186/s12870-024-05262-7 (PMC11177404; doi:10.1186/s12870-024-05262-7)
Supplement: Supplementary file 1 — Additional file 1: Table S1. Primer information of 15 GhEPF/EPFL and Ubiquitin7 for qRT-PCR experiments. Table S2. Identification and physiochemical characteristic analysis of cotton EPF/EPFL genes. [file 12870_2024_5262_MOESM1_ESM.docx]

Additional file 1 Table S1 Primer information of 15 *GhEPF/EPFL* and *Ubiquitin7* for qRT-PCR experiments

| Primer name | Forward sequences | Reverse sequences |
| --- | --- | --- |
| *Ubiquitin7*（*UB7*） | CTCCGAGAACGTCATCACCG | TGGAGC CGTACTGGAACTGG |
| *GhEPF4* (*GH_A03G0330*) | CAACCACATGCCAACGAGC | ACTTTGAGGCACACCGTGG |
| *GhEPF10* (*GH_A05G2141*) | TCCTTCAAGCGTCGTGTTGT | TCAACACGTTCCCAGCTCAT |
| *GhEPF13* (*GH_A07G2235*) | ATTGGATCCACAGCCCCTAC | CTTGCTCAGCTCTGCATTTGT |
| *GhEPF14* (*GH_A09G2397*) | TGGGTCAGTGGTGTTGAACC | AACGCCAAGCTTCAGGGTAG |
| *GhEPF17* (*GH_A11G0525*) | TCCCCAGAGAGCTTGCCATA | TTCTCTCACATCTTGGCGGC |
| *GhEPF20* (*GH_A11G1491*) | GCAGCGCATGCAATACAAGG | AAGTGCAGGTTGGAGCTGTG |
| *GhEPF23* (*GH_D02G2237*) | CGCCACCACATCTTAGTTGC | AACCTGGTTGAATCGGGACG |
| *GhEPF25* (*GH_D03G0395*) | ACCTCACTTGTTTCTCCCCT | ACATCTGTTGTGGCAGCTTG |
| *GhEPF26* (*GH_D03G1641*) | ACCCACAAATCCCACTTGCT | GCCCTTGGATGTCAACAGGT |
| *GhEPF28* (*GH_D05G0160*) | AAACAGTTCTGGGTTCGAGGC | CCCACATTTGCATTTCCAGGC |
| *GhEPF29* (*GH_D05G2173*) | ACCTTTGCTACTCTTCACCTTCTT | ACTTGCTCAGCTCTGCATTTG |
| *GhEPF39* (*GH_D10G1648*) | CTCTTTCTCGATTCGGCAGC | AGGTGAGCAGTTTCCACACTT |
| *GhEPF40* (*GH_D11G2297*) | CATGGCAGTCCAAGTGCCTA | AAAAGTGATCATCGCAGCGG |
| *GhEPF41* (*GH_D11G0547*) | TCCCCAGAGAGCTTGCCATA | TTCTCTCACATCTTGGCGGC |
| *GhEPF43* (*GH_D11G1520*) | CAGCGCATGCAATACAAGGA | TAAGTGCAGGTTGGAGCTGT |
