# Supplementary material for: Genome-wide analyses of member identification, expression pattern, and protein–protein interaction of EPF/EPFL gene family in Gossypium
Source: BMC Plant Biol. 2024 Jun 14;24:554. doi: 10.1186/s12870-024-05262-7 (PMC11177404; doi:10.1186/s12870-024-05262-7)
Supplement: Supplementary file 2 — Additional file 2: Figure S1. The conserved peptides among the EPF/EPFL proteins. Figure S2. The information of 8 conserved motifs. [file 12870_2024_5262_MOESM2_ESM.docx]

Additional file 1: Table S2 Identification and physiochemical characteristic analysis of cotton EPF/EPFL genes

| Gene Name | Gene ID | Protein Length (aa) | Molecular Weight (kDa) | Isoelectric Point | Instability Index | Subcellular Prediction | Genome Location |
| --- | --- | --- | --- | --- | --- | --- | --- |
| *GaEPF1* | *Ga01G2467* | 135 | 14795.20 | 8.92 | 44.96 | chlo | Chr01: 108287053-108288318+ |
| *GaEPF2* | *Ga01G0194* | 141 | 15726.42 | 9.02 | 71.66 | chlo | Chr01: 1392565-1392990- |
| *GaEPF3* | *Ga02G0388* | 118 | 13153.98 | 8.24 | 58.07 | extr | Chr02: 5207547-5207985- |
| *GaEPF4* | *Ga02G0311* | 101 | 11528.24 | 9.35 | 49.63 | chlo | Chr02: 3244046-3244415+ |
| *GaEPF5* | *Ga03G0719* | 115 | 12715.05 | 8.05 | 45.61 | extr | Chr03: 11087565-11088069- |
| *GaEPF6* | *Ga03G2396* | 101 | 11150.84 | 5.63 | 78.50 | extr | Chr03: 132537547-132537932- |
| *GaEPF7* | *Ga05G1447* | 135 | 14844.98 | 8.43 | 66.65 | chlo | Chr05: 12829678-12830176+ |
| *GaEPF8* | *Ga05G0325* | 131 | 14377.20 | 7.57 | 40.84 | chlo | Chr05: 2936977-2937652+ |
| *GaEPF9* | *Ga05G2214* | 134 | 15338.86 | 9.16 | 56.56 | extr | Chr05: 20563768-20564374- |
| *GaEPF10* | *Ga05G2904* | 120 | 13122.58 | 8.04 | 45.63 | vacu | Chr05: 30508555-30509206+ |
| *GaEPF11* | *Ga05G0162* | 107 | 12141.16 | 9.51 | 50.83 | extr | Chr05: 1520736-1521163+ |
| *GaEPF12* | *Ga06G0762* | 119 | 13544.99 | 9.76 | 40.61 | vacu | Chr06: 14076503-14077231+ |
| Gene Name | Gene ID | Protein Length (aa) | Molecular Weight (kDa) | Isoelectric Point | Instability Index | Subcellular Prediction | Genome Location |
| *GaEPF13* | *Ga07G2282* | 108 | 12325.24 | 9.17 | 60.07 | chlo | Chr07: 91592341-91592921- |
| *GaEPF14* | *Ga07G1909* | 110 | 12368.37 | 9.00 | 57.48 | extr | Chr07: 59303448-59304107+ |
| *GaEPF15* | *Ga09G2500* | 137 | 15400.10 | 9.35 | 75.98 | E.R. | Chr09: 82360883-82361483- |
| *GaEPF16* | *Ga10G2468* | 141 | 15180.98 | 6.80 | 58.42 | extr | Chr10: 123168995-123169505+ |
| *GaEPF17* | *Ga10G2315* | 105 | 11762.78 | 9.10 | 60.77 | chlo | Chr10: 119347456-119347855+ |
| *GaEPF18* | *Ga10G1388* | 110 | 12025.90 | 9.84 | 59.18 | chlo | Chr10: 66006787-66007236- |
| *GaEPF19* | *Ga11G3559* | 127 | 14394.66 | 9.56 | 46.35 | extr | Chr11: 119639253-119639803- |
| *GaEPF20* | *Ga11G2536* | 109 | 12437.35 | 9.10 | 72.44 | chlo | Chr11: 106818375-106818953- |
| *GaEPF21* | *Ga11G1658* | 127 | 14382.62 | 9.51 | 45.25 | nucl | Chr11: 69495273-69496269- |
| *GaEPF22* | *Ga11G1763* | 129 | 14409.61 | 9.84 | 46.39 | mito | Chr11: 80602707-80603262- |
| *GaEPF23* | *Ga12G2180* | 128 | 14717.09 | 9.69 | 32.13 | chlo | Chr12: 66292026-66292613+ |
| *GaEPF24* | *Ga14G1483* | 122 | 13681.15 | 9.72 | 48.82 | chlo | tig00013782: 98446-99000- |
| *GrEPF1* | *rna-XM_012636751.1* | 110 | 12271.21 | 9.38 | 67.46 | chlo | Gr01: 46534270-46535469- |
| Gene Name | Gene ID | Protein Length (aa) | Molecular Weight (kDa) | Isoelectric Point | Instability Index | Subcellular Prediction | Genome Location |
| *GrEPF2* | *rna-XM_012613587.1* | 141 | 15536.16 | 8.59 | 67.21 | extr | Gr02: 1201234-1202079- |
| *GrEPF3* | *rna-XM_012614351.1* | 119 | 13257.11 | 8.24 | 59.29 | extr | Gr03: 4812214-4813146- |
| *GrEPF4* | *rna-XM_012617136.1* | 101 | 11479.17 | 9.38 | 48.41 | chlo | Gr03: 3295052-3295757+ |
| *GrEPF5* | *rna-XM_012616271.1* | 144 | 15937.55 | 9.28 | 45.61 | chlo | Gr03: 42060860-42062910- |
| *GrEPF6* | *rna-XM_012626908.1* | 116 | 12772.02 | 8.60 | 40.20 | extr | Gr05: 8671637-8672634- |
| *GrEPF7* | *rna-XM_012624686.1* | 101 | 11200.90 | 5.63 | 74.15 | extr | Gr05: 61401682-61402843- |
| *GrEPF8* | *rna-XM_012631742.1* | 110 | 12239.35 | 9.75 | 72.47 | chlo | Gr06: 48827847-48828686- |
| *GrEPF9* | *rna-XM_012636579.1* | 129 | 14405.66 | 9.82 | 47.56 | mito | Gr07: 27762314-27763652+ |
| *GrEPF10* | *rna-XM_012633780.1* | 127 | 14241.47 | 9.32 | 48.65 | extr | Gr07: 4093479-4095917+ |
| *GrEPF11* | *rna-XM_012635459.1* | 111 | 12602.54 | 9.12 | 76.67 | chlo | Gr07: 13867323-13868259+ |
| *GrEPF12* | *rna-XM_012590025.1* | 134 | 15310.81 | 9.18 | 51.44 | extr | Gr09: 17432568-17434760+ |
| *GrEPF13* | *rna-XM_012585058.1* | 123 | 13577.16 | 8.35 | 51.58 | vacu | Gr09: 24521893-24523018+ |
| *GrEPF14* | *rna-XM_012585253.1* | 123 | 13796.82 | 8.87 | 38.13 | extr | Gr09: 2435436-2438645+ |
| Gene Name | Gene ID | Protein Length (aa) | Molecular Weight (kDa) | Isoelectric Point | Instability Index | Subcellular Prediction | Genome Location |
| *GrEPF15* | *rna-XM_012585172.1* | 135 | 14932.10 | 8.42 | 68.80 | chlo | Gr09: 10789160-10791556+ |
| *GrEPF16* | *rna-XM_012593163.1* | 122 | 13653.84 | 9.40 | 39.35 | chlo | Gr10: 12667866-12668682+ |
| *GrEPF17* | *rna-XM_012594968.1* | 134 | 15003.84 | 9.83 | 47.77 | extr | Gr10: 2419614-2429157+ |
| *GrEPF18* | *rna-XM_012598611.1* | 108 | 12077.11 | 9.02 | 57.40 | extr | Gr11: 8457480-8458183+ |
| *GrEPF19* | *rna-XM_012602375.1* | 110 | 12065.93 | 9.69 | 58.50 | chlo | Gr11: 34450123-34451189- |
| *GrEPF20* | *rna-XM_012610556.1* | 87 | 9760.13 | 9.10 | 59.44 | nucl | NW_012160881.1: 1107602-1108896- |
| *GhEPF1* | *Ghir_A01G001760.1* | 124 | 13609.08 | 9.18 | 65.21 | chlo | GhA01: 1540608-1541021- |
| *GhEPF2* | *Ghir_A02G014280.1* | 119 | 13253.12 | 8.24 | 57.67 | extr | GhA02: 97532173-97533169- |
| *GhEPF3* | *Ghir_A02G006430.1* | 94 | 10464.63 | 8.78 | 47.87 | chlo | GhA02: 10046318-10046822- |
| *GhEPF4* | *Ghir_A03G003070.1* | 143 | 15809.46 | 9.18 | 44.21 | chlo | GhA03: 4648642-4650548+ |
| *GhEPF5* | *Ghir_A03G020070.1* | 101 | 11150.84 | 5.63 | 84.76 | extr | GhA03: 109878004-109878460- |
| *GhEPF6* | *Ghir_A05G014050.1* | 135 | 14844.98 | 8.43 | 66.65 | chlo | GhA05: 13094991-13097281+ |
| *GhEPF7* | *Ghir_A05G027780.1* | 148 | 16371.30 | 9.39 | 66.15 | mito | GhA05: 30092619-30093451+ |
| Gene Name | Gene ID | Protein Length (aa) | Molecular Weight (kDa) | Isoelectric Point | Instability Index | Subcellular Prediction | Genome Location |
| *GhEPF8* | *Ghir_A05G001640.1* | 122 | 13830.24 | 9.63 | 55.99 | extr | GhA05: 1629976-1630839+ |
| *GhEPF9* | *Ghir_A05G003180.1* | 109 | 11952.57 | 7.57 | 34.02 | chlo | GhA05: 3063992-3065324+ |
| *GhEPF10* | *Ghir_A05G021880.1* | 118 | 13517.69 | 9.10 | 53.12 | extr | GhA05: 21159931-21160538+ |
| *GhEPF11* | *Ghir_A06G007490.1* | 122 | 13761.04 | 9.74 | 40.89 | chlo | GhA06: 17709488-17710212+ |
| *GhEPF12* | *Ghir_A07G017060.1* | 110 | 12368.37 | 9.00 | 57.48 | extr | GhA07: 49217429-49218088+ |
| *GhEPF13* | *Ghir_A07G021240.1* | 108 | 12325.24 | 9.17 | 60.07 | chlo | GhA07: 92030482-92031425- |
| *GhEPF14* | *Ghir_A09G023330.1* | 110 | 12252.35 | 9.75 | 70.03 | chlo | GhA09: 79394793-79395716- |
| *GhEPF15* | *Ghir_A10G005500.1* | 129 | 13886.55 | 5.53 | 55.13 | extr | GhA10: 6064920-6065430- |
| *GhEPF16* | *Ghir_A10G011650.1* | 110 | 12035.94 | 9.84 | 59.18 | chlo | GhA10: 47661717-47662983+ |
| *GhEPF17* | *Ghir_A11G005520.1* | 127 | 14326.58 | 9.44 | 50.25 | chlo | GhA11: 4925769-4928400+ |
| *GhEPF18* | *Ghir_A11G022460.1* | 129 | 14413.60 | 9.84 | 46.98 | mito | GhA11: 60175221-60175988- |
| *GhEPF19* | *Ghir_A11G021600.1* | 127 | 14224.48 | 9.30 | 53.90 | extr | GhA11: 48558439-48559512- |
| *GhEPF20* | *Ghir_A11G015120.1* | 108 | 12864.89 | 9.58 | 70.92 | mito | GhA11: 17199680-17201891+ |
| Gene Name | Gene ID | Protein Length (aa) | Molecular Weight (kDa) | Isoelectric Point | Instability Index | Subcellular Prediction | Genome Location |
| *GhEPF21* | *Ghir_A12G008890.1* | 128 | 14716.15 | 9.86 | 30.62 | chlo | GhA12: 45682442-45683518- |
| *GhEPF22* | *Ghir_D01G001740.1* | 122 | 13238.58 | 8.40 | 60.68 | extr | GhD01: 1283281-1283694- |
| *GhEPF23* | *Ghir_D02G021470.1* | 101 | 11200.90 | 5.63 | 74.15 | extr | GhD02: 66949770-66951595- |
| *GhEPF24* | *Ghir_D02G006820.1* | 110 | 12098.27 | 8.76 | 43.65 | extr | GhD02: 9472712-9473219- |
| *GhEPF25* | *Ghir_D03G003940.1* | 118 | 13064.85 | 8.25 | 53.68 | extr | GhD03: 4907803-4908736- |
| *GhEPF26* | *Ghir_D03G015940.1* | 143 | 15866.47 | 9.28 | 46.45 | extr | GhD03: 48534329-48536213- |
| *GhEPF27* | *Ghir_D05G003310.1* | 131 | 14551.63 | 8.62 | 38.07 | extr | GhD05: 2798117-2799352+ |
| *GhEPF28* | *Ghir_D05G001840.1* | 107 | 12113.11 | 9.51 | 51.98 | extr | GhD05: 1609293-1610212+ |
| *GhEPF29* | *Ghir_D05G021800.1* | 118 | 13501.69 | 9.11 | 51.03 | extr | GhD05: 19124880-19126357+ |
| *GhEPF30* | *Ghir_D05G013790.1* | 135 | 14820.96 | 8.12 | 68.29 | chlo | GhD05: 11862966-11864436+ |
| *GhEPF31* | *Ghir_D05G027790.1* | 148 | 16494.59 | 8.86 | 61.98 | mito | GhD05: 26740476-26741260+ |
| *GhEPF32* | *Ghir_D06G007930.1* | 122 | 13631.75 | 9.40 | 39.47 | chlo | GhD06: 13617694-13618461+ |
| *GhEPF33* | *Ghir_D07G021420.1* | 108 | 12234.13 | 9.19 | 63.60 | chlo | GhD07: 53808960-53810201- |
| Gene Name | Gene ID | Protein Length (aa) | Molecular Weight (kDa) | Isoelectric Point | Instability Index | Subcellular Prediction | Genome Location |
| *GhEPF34* | *Ghir_D07G017550.2* | 110 | 12370.33 | 9.10 | 58.63 | chlo | GhD07: 32294476-32295487+ |
| *GhEPF35* | *Ghir_D07G017550.1* | 87 | 9779.14 | 8.95 | 66.71 | nucl | GhD07: 32294137-32295446+ |
| *GhEPF36* | *Ghir_D09G022560.1* | 161 | 18049.99 | 9.80 | 65.04 | E.R. | GhD09: 50402404-50403359- |
| *GhEPF37* | *Ghir_D10G006330.1* | 141 | 15323.06 | 6.87 | 55.63 | chlo | GhD10: 5973676-5974186- |
| *GhEPF38* | *Ghir_D10G008200.1* | 105 | 11801.80 | 9.02 | 58.76 | chlo | GhD10: 8861770-8862169+ |
| *GhEPF39* | *Ghir_D10G016030.1* | 110 | 12096.04 | 9.84 | 53.82 | chlo | GhD10: 35250108-35251147- |
| *GhEPF40* | *Ghir_D11G022820.1* | 127 | 14208.48 | 9.30 | 52.38 | extr | GhD11: 33884307-33885282+ |
| *GhEPF41* | *Ghir_D11G005470.1* | 127 | 14241.47 | 9.32 | 48.65 | extr | GhD11: 4565312-4566616+ |
| *GhEPF42* | *Ghir_D11G021940.1* | 129 | 14433.67 | 9.86 | 47.64 | mito | GhD11: 29322003-29323536+ |
| *GhEPF43* | *Ghir_D11G015200.1* | 116 | 13326.61 | 9.05 | 70.67 | extr | GhD11: 14774663-14775237+ |
| *GhEPF44* | *Ghir_D12G008540.1* | 128 | 14708.03 | 9.91 | 35.64 | chlo | GhD12: 25793187-25794180- |
| *GbEPF1* | *Gbar_A01G001510.1* | 124 | 13609.08 | 9.18 | 65.21 | chlo | GbA01: 1303686-1304099- |
| *GbEPF2* | *Gbar_A02G013950.1* | 119 | 13244.11 | 8.24 | 57.91 | extr | GbA02: 90031185-90032256- |
| Gene Name | Gene ID | Protein Length (aa) | Molecular Weight (kDa) | Isoelectric Point | Instability Index | Subcellular Prediction | Genome Location |
| *GbEPF3* | *Gbar_A02G006250.1* | 94 | 10448.57 | 8.85 | 45.82 | chlo | GbA02: 9913301-9913805- |
| *GbEPF4* | *Gbar_A03G003050.1* | 144 | 15880.54 | 9.18 | 43.39 | chlo | GbA03: 3871165-3873039+ |
| *GbEPF5* | *Gbar_A03G020140.1* | 101 | 11150.84 | 5.63 | 78.50 | extr | GbA03: 102190887-102191272- |
| *GbEPF6* | *Gbar_A05G002710.1* | 131 | 14352.19 | 6.93 | 44.13 | chlo | GbA05: 2683427-2684224+ |
| *GbEPF7* | *Gbar_A05G021160.1* | 134 | 15326.81 | 9.16 | 53.29 | extr | GbA05: 20306608-20307214+ |
| *GbEPF8* | *Gbar_A05G001660.1* | 117 | 13150.36 | 9.73 | 60.03 | chlo | GbA05: 1455175-1455632+ |
| *GbEPF9* | *Gbar_A05G026790.1* | 108 | 11804.00 | 8.78 | 46.24 | extr | GbA05: 28745111-28745763+ |
| *GbEPF10* | *Gbar_A05G013450.1* | 119 | 13084.04 | 6.80 | 60.70 | chlo | GbA05: 12469545-12470043+ |
| *GbEPF11* | *Gbar_A06G007670.1* | 122 | 13761.04 | 9.74 | 40.89 | chlo | GbA06: 17777878-17778604+ |
| *GbEPF12* | *Gbar_A07G017070.1* | 110 | 12368.37 | 9.00 | 57.48 | extr | GbA07: 47040478-47041137+ |
| *GbEPF13* | *Gbar_A07G020980.1* | 108 | 12325.24 | 9.17 | 60.07 | chlo | GbA07: 87307199-87308169- |
| *GbEPF14* | *Gbar_A09G023510.1* | 108 | 12025.04 | 9.63 | 68.65 | chlo | GbA09: 75267181-75267769- |
| *GbEPF15* | *Gbar_A10G012630.1* | 110 | 12035.94 | 9.84 | 59.18 | chlo | GbA10: 47397926-47399029+ |
| Gene Name | Gene ID | Protein Length (aa) | Molecular Weight (kDa) | Isoelectric Point | Instability Index | Subcellular Prediction | Genome Location |
| *GbEPF16* | *Gbar_A10G007880.1* | 105 | 11762.78 | 9.10 | 60.77 | chlo | GbA10: 11342757-11343155- |
| *GbEPF17* | *Gbar_A10G006140.1* | 129 | 13886.55 | 5.53 | 55.13 | extr | GbA10: 6224552-6225062- |
| *GbEPF18* | *Gbar_A11G014750.1* | 109 | 12437.35 | 9.10 | 72.44 | chlo | GbA11: 16301889-16302857+ |
| *GbEPF19* | *Gbar_A11G021820.1* | 129 | 14413.60 | 9.84 | 46.98 | mito | GbA11: 55056648-55057203- |
| *GbEPF20* | *Gbar_A11G020910.1* | 127 | 14238.50 | 9.30 | 52.38 | extr | GbA11: 44480810-44481738- |
| *GbEPF21* | *Gbar_A11G005160.1* | 127 | 14326.58 | 9.44 | 50.25 | chlo | GbA11: 4561455-4564159+ |
| *GbEPF22* | *Gbar_A12G009230.1* | 115 | 13329.62 | 9.84 | 36.07 | chlo | GbA12: 49403323-49403910- |
| *GbEPF23* | *Gbar_D01G001690.1* | 141 | 15585.28 | 6.39 | 52.76 | E.R. | GbD01: 1293786-1295096- |
| *GbEPF24* | *Gbar_D02G007180.1* | 116 | 12741.99 | 8.60 | 43.98 | extr | GbD02: 9702768-9703331- |
| *GbEPF25* | *Gbar_D02G022070.1* | 101 | 11200.90 | 5.63 | 74.15 | extr | GbD02: 64808882-64809267- |
| *GbEPF26* | *Gbar_D03G015410.1* | 130 | 14408.76 | 9.28 | 44.97 | extr | GbD03: 47394354-47395627- |
| *GbEPF27* | *Gbar_D03G003910.1* | 118 | 13105.90 | 8.25 | 53.04 | extr | GbD03: 4800220-4800859- |
| *GbEPF28* | *Gbar_D05G027630.1* | 112 | 12409.81 | 8.39 | 53.48 | vacu | GbD05: 26405667-26406282+ |
| Gene Name | Gene ID | Protein Length (aa) | Molecular Weight (kDa) | Isoelectric Point | Instability Index | Subcellular Prediction | Genome Location |
| *GbEPF29* | *Gbar_D05G001690.1* | 107 | 12113.11 | 9.51 | 51.98 | extr | GbD05: 1488974-1489601+ |
| *GbEPF30* | *Gbar_D05G003140.1* | 106 | 12122.01 | 8.97 | 37.57 | extr | GbD05: 2656633-2657308+ |
| *GbEPF31* | *Gbar_D06G007980.1* | 122 | 13631.75 | 9.40 | 39.47 | chlo | GbD06: 13023227-13023994+ |
| *GbEPF32* | *Gbar_D06G002710.1* | 100 | 11129.06 | 9.40 | 48.13 | chlo | GbD06: 2660444-2660933+ |
| *GbEPF33* | *Gbar_D07G017630.1* | 110 | 12370.33 | 9.10 | 58.63 | chlo | GbD07: 31997480-31998139+ |
| *GbEPF34* | *Gbar_D07G021630.1* | 108 | 12185.14 | 9.47 | 64.01 | chlo | GbD07: 50923589-50924438- |
| *GbEPF35* | *Gbar_D09G023170.1* | 161 | 18049.05 | 9.80 | 67.17 | E.R. | GbD09: 49043331-49044243- |
| *GbEPF36* | *Gbar_D10G007920.1* | 105 | 11801.80 | 9.02 | 58.76 | chlo | GbD10: 8336223-8336621+ |
| *GbEPF37* | *Gbar_D10G006070.1* | 116 | 12452.70 | 5.91 | 53.85 | nucl | GbD10: 5572358-5572954- |
| *GbEPF38* | *Gbar_D10G015740.1* | 110 | 12096.04 | 9.84 | 53.82 | chlo | GbD10: 34702574-34703877- |
| *GbEPF39* | *Gbar_D11G022090.1* | 129 | 14433.67 | 9.86 | 47.64 | mito | GbD11: 28863045-28863601+ |
| *GbEPF40* | *Gbar_D11G015570.1* | 109 | 12335.21 | 9.14 | 75.30 | chlo | GbD11: 14478853-14479865+ |
| *GbEPF41* | *Gbar_D11G005490.1* | 127 | 14241.47 | 9.32 | 48.65 | extr | GbD11: 4393301-4394808+ |
| Gene Name | Gene ID | Protein Length (aa) | Molecular Weight (kDa) | Isoelectric Point | Instability Index | Subcellular Prediction | Genome Location |
| *GbEPF42* | *Gbar_D11G023020.1* | 127 | 14181.45 | 9.30 | 53.55 | extr | GbD11: 33186805-33188117+ |
| *GbEPF43* | *Gbar_D05G040100.1* | 134 | 15310.81 | 9.18 | 51.44 | extr | Scaffold2259: 37916-40257+ |
| *GbEPF44* | *Gbar_Scaffold2479G000010.1* | 138 | 15625.42 | 9.64 | 39.47 | plas | Scaffold2479: 3821-4426+ |
